# Structural and optical properties of spin coated ZnTTBPc thin films for optoelectronics via DFT and experiments

**DOI:** 10.1038/s41598-025-21726-8

**Published:** 2025-10-17

**Authors:** Mostafa Saad Ebied, Sahar Elnobi, Amr Attia Abuelwafa

**Affiliations:** 1 Nano and Thin Film Lab. Physics Department, Faculty of Science, South Valley University, Qena, 83523 Egypt; 2 Physics Department, Faculty of Science, South Valley University, Qena, 83523 Egypt

**Keywords:** ZnTTBPc, DFT, HOMO-LUMO, NLO properties, Optoelectronics, Spin coating, Thin films, Materials science, Optics and photonics

## Abstract

The present research provides a comprehensive theoretical and experimental investigation into the structural and optical properties of zinc 2, 9, 16, 23-tetra-tert-butyl-29 H,31 H-phthalocyanine (ZnTTBPc). Density functional theory (DFT) calculations at the B3LYP/6-31G (d, p) level, were utilized to optimize the molecular structure, analyze Mulliken charges, generate a molecular electrostatic potential map, and compute frontier molecular orbital energies of ZnTTBPc molecule. The nonlinear optical properties, including polarizability and hyperpolarizability, were also determined, confirming the molecule’s applicability for nonlinear optical applications. Experimentally, spin-coated ZnTTBPc thin films were fabricated on quartz substrates. Structural characterization using X-ray diffraction and Raman spectroscopy indicated the amorphous nature of the thin films, whereas field emission scanning electron microscopy demonstrated a uniform, nanostructured morphology. Optical properties, including absorption coefficient, refractive index, and extinction coefficient, were analyzed over a wide spectral range (200–2000 nm), demonstrating the presence of direct optical transitions. The energy band gaps corresponding to the Q and B bands were obtained from Tauc plot analysis, with values of 1.42 and 2.25 eV respectively. The dispersion parameters and dielectric properties were evaluated using a single oscillator model. The nonlinear optical parameters such as the third-order nonlinear susceptibility, nonlinear refractive index and nonlinear absorption coefficient were calculated using semi-empirical relations. The results demonstrate that ZnTTBPc thin films possess excellent optical and nonlinear properties, making them highly promising candidates for advanced optoelectronic and photonic devices. This integrated theoretical and experimental approach underscores the importance of molecular design and fabrication techniques in optimizing materials for cutting-edge applications.

**Supplementary Information:**

The online version contains supplementary material available at 10.1038/s41598-025-21726-8.

## Introduction

Phthalocyanines (Pcs) are a large aromatic compounds characterized by their planar, two-dimensional structure composed of four isoindole units interconnected by nitrogen atoms. The delocalized π-electrons in the Pc structure are responsible for their strong absorption in the visible spectrum region^1^. Phthalocyanines (Pcs) have attracted considerable interest owing to their unique chemical and physical properties, enabling their use in a wide range of applications. These materials have been utilized as photosensitive optical and electronic materials in solar cells, photodynamic therapy, diagnostic agents, light harvesting, nonlinear absorption, optical storage devices, and sensors^2–5^. The functionality of Pcs-based materials can be optimized by fine-tuning their absorption bands to target certain regions of the electromagnetic spectrum, hence improving their performance for practical applications^4–7^. Metallophthalocyanines (MPcs), derived by incorporating divalent metal atoms like Zn, Cu, Co, or Ni into the phthalocyanine core, provide additional structural flexibility and tunability. This modification influences their crystallinity, morphology, and electronic and optical properties, thereby enabling precise optimization for particular applications^8^. MPcs exhibit strong absorption in the 500–740 nm wavelength range due to the Q-band, which is characterized by high molar extinction coefficients, making them exemplary materials for light-harvesting applications^9^. The Q-band’s characteristics, including its position, bandwidth, and symmetry, can be further tailored through the incorporation of various substituents.

Several studies have investigated the fundamental properties of various MPcs, with zinc phthalocyanine (ZnPc) emerging as one of the most extensively studied and promising materials^10–14^. In addition, ZnPc exhibits unique photoelectric properties due to its distinct electronic structures^3,15,16^ compared to other metal-substituted phthalocyanines. The functionality of ZnPc can be further tailored through peripheral substitutions, enhancing their solubility in organic solvents. For example, the addition of four tert-butyl (tBu) groups to the periphery benzene rings of zinc (II) phthalocyanine yields zinc 2,9,16,23-tetra-tert-butyl-29 H,31 H-phthalocyanine (ZnTTBPc). These tert-butyl groups provide a three-dimensional structure to the otherwise planar ZnPc molecules, enhancing solubility, chemical stability, and lightfastness. Additionally, tert-butyl substitutions, being bulky, inhibit coplanar π-π interactions between phthalocyanine molecules, which affects the crystalline polymorphic state of the MPcs and enhances their functional properties^17^.

ZnTTBPc has been investigated in several prior studies for its photophysical, structural, and sensor-related properties, A spin coated ZnTTBPc thin films^18^, have been investigated as sensitive materials for volatile organic compounds (VOCs) by analyzing their optical response to these compounds. The ZnTTBPc-based optical sensor demonstrated its efficiency to distinguish among diverse VOCs. Rejitha et al. ^17^, investigated the annealing effects on the optical, electrical and surface morphological properties of thermal evaporated ZnTTBPc thin films. The impact of solvent polarity on the (UV–vis) absorption and fluorescence emission spectroscopies of ZnTTBPc has been studied^19^. The third order nonlinear optical (NLO) coefficients of thermally evaporated β -phase ZnTTBPc thin films using ps laser pulses has been investigated^20^, demonstrating potential for nonlinear optical device applications.

Characterization techniques, including infrared and Raman spectroscopies have proven invaluable for analyzing MPcs-containing thin films. Despite their utility, spectrum interpretation for phthalocyanines is more complex than that for structurally similar compounds such as porphyrins, which limits the effectiveness of vibrational methods in exploring structural variations^21–23^. To address this challenge, computational approaches offer a more profound understanding of the electronic structure of these materials. Among the various computational techniques, density functional theory (DFT) has emerged as the most widely used method due to its high accuracy-to-effort ratio and versatility in predicting the properties of diverse compounds^24–27^. DFT provides comprehensive insights into the electronic and photophysical mechanisms underlying the behavior of phthalocyanines^28–31^.

The originality of this study belong to in the combined theoretical and experimental analysis of ZnTTBPc which has not been thoroughly characterized in spin-coated thin film form for optoelectronic applications. This study provides a comprehensive perspective on the influence of molecular structure on device-relevant properties by integrating density functional theory with an in-depth optical and nonlinear characterization of spin-coated ZnTTBPc thin films. Adding tert-butyl groups makes the material more soluble and breaks up π–π stacking, which makes it easier to form films and less likely to clump together. These are important for getting uniform, high-quality active layers. The thorough optical analysis across a wide wavelength range (200–2000 nm), combined with nonlinear optical parameters derived from semi-empirical methods, yields essential information regarding the applicability of ZnTTBPc for optical switching, nonlinear photonics, and broadband light harvesting.

## Materials and methods

### Computational calculations

All density functional theory (DFT) calculations were performed using the Gaussian 09 software package^32^ .The ground-state geometry of the ZnTTBPc molecule was optimized in the gas phase at the B3LYP/6-31G(d, p) level of theory^33^. Following optimization, vibrational frequency analysis was conducted at the same theoretical level confirming that the optimized structure corresponds to a local minima on the potential energy surface (i.e., no imaginary frequency was observed). The energy values of the HOMO, LUMO, and HOMO–LUMO bandgap were computed from the optimized geometry of the ground state of the ZnTTBPc molecule. The NLO properties of ZnTTBPc molecule such as polarizability ($\:{\alpha\:}_{p}$) and hyperpolarizability ($\:\beta\:$) were computed by CAM-B3LYP/6-31G(d, p) .TD-DFT calculations (CAM-B3LYP/6-31G(d, p) method) were conducted to simulate the UV–vis absorption spectra and evaluate the properties of the excited state of the ZnTTBPc molecule.

### Preparation and characterization of ZnTTBPc thin film

**Scheme 1:**
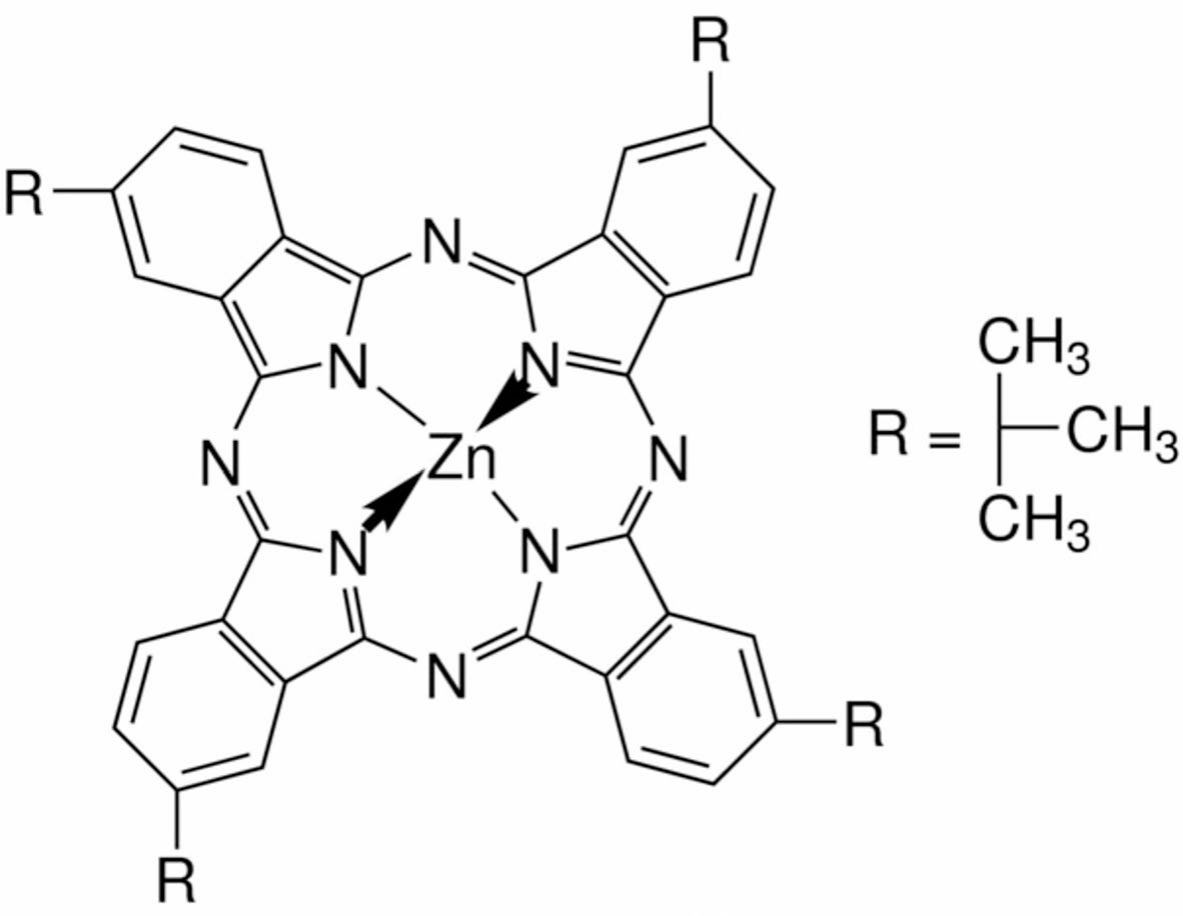
Schematic diagram of ZnTTBPc molecule.

ZnTTBPc with a purity of 96% was purchased from Sigma-Aldrich and used without further purification. It was dissolved in chloroform at a concentration of 25 mg/mL (**see** Scheme 1). This mixture was then applied to pre-cleaned quartz substrates using a spin coating technique with a spinning speed of 2500 rpm for duration of 30 s. The film was exposed to heating at 50 °C for 60 s to expedite solvent evaporation. The thickness of thin coatings was measured using a surface profilometer Alpha-Step IQ surface (~ 100 nm). XRD patterns of powder and thin film of ZnTTBPc were obtained by employing a Rigaku RINT 2100 diffractometer. By using FE-SEM (JEOL model JSM-7001 F), the morphology of an as-deposited ZnTTBPc thin film was investigated. The Raman shift for both the powder and thin film of ZnTTBPc at room temperature was measured using a Jasco NRS-2100 spectrometer. We measured the optical transmittance T (λ) and reflectance R (λ) of a thin film within the wavelength range of 200 to 2000 nm using a spectrophotometer (JASCO V-670 UV-Vis-NIR model).

## Results and discussions

### Computational study of ZnTTBPc molecule

#### Geometry optimization

**Fig. 1:**
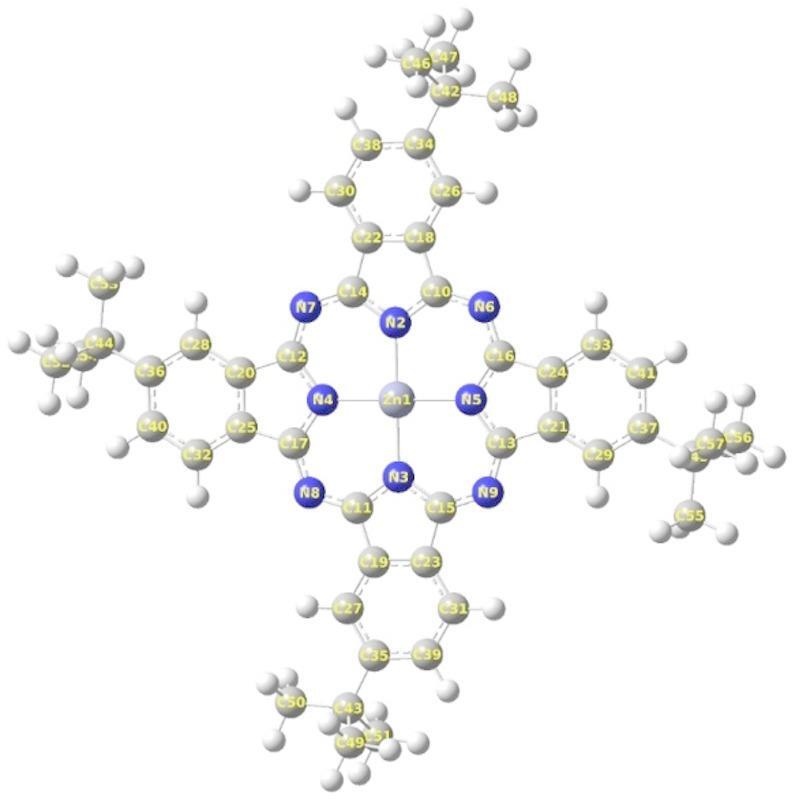
Optimized structure of ZnTTBPc molecule.

The ZnTTBPc structure (Scheme 1) includes a planar core (ZnPc) and bending side groups (tert-butyl). The initial structure of ZnTTBPc molecule used in this study was downloaded from ChemSpider website (ChemSpider ID 3346810). Table 1 displays computationally calculated structural characteristics such as bond lengths, bond angles, and torsion angles (full parameters are included in Table (S1, S2 and S3) of the supplementary information file). The geometry of the ZnTTBPc molecule optimized using the B3LYP/6-31G(d, p) approach is shown in Fig. 1. The bonding distance between the Zinc atom in the phthalocyanine core and the four nitrogen atoms around it (Zn1-N2, Zn1-N3, Zn1-N4, Zn1-N5) is 1.989 Å, demonstrating that the Zinc atom is completely positioned in the phthalocyanine core at an angle of about 90. The computed distances between N-C bonds vary from 1.3302 to 1.3738 Å. Dihedral angles approaching 0 or 180 in the optimized geometry indicate that the phthalocyanine ring is planar. The optimized cartesian coordinates of our calculated molecule are presented in the supplementary information file (see Table S4).

**Table 1:** Theoretically obtained some bond lengths (Å), bond angles (^o^), and dihedral angles (^o^) of ZnTTBPc molecule.

Atom groups	Bond Lengths (Å)	Atom groups	Bond angles (^o^)	Atom groups	Dihedral angles (^o^)
Zn1-N2	1.99	N2-Zn1-N4	90	N4-Zn1-N2-C10	179.9997
Zn1-N3	1.99	N2-Zn1-N5	90	N4-Zn1-N2-C14	-0.001
Zn1-N4	1.99	N3-Zn1-N4	90	N5-Zn1-N2-C10	0.001
Zn1-N5	1.99	N3-Zn1-N5	90	N5-Zn1-N2-C14	-179.9997
N2-C14	1.3733	C12-N4-C17	109.3497		
N6-C10	1.3302				
C10-C18	1.4612				
C26-H58	1.0828				

#### Frontier molecular orbital and electronic properties

**Fig. 2:**
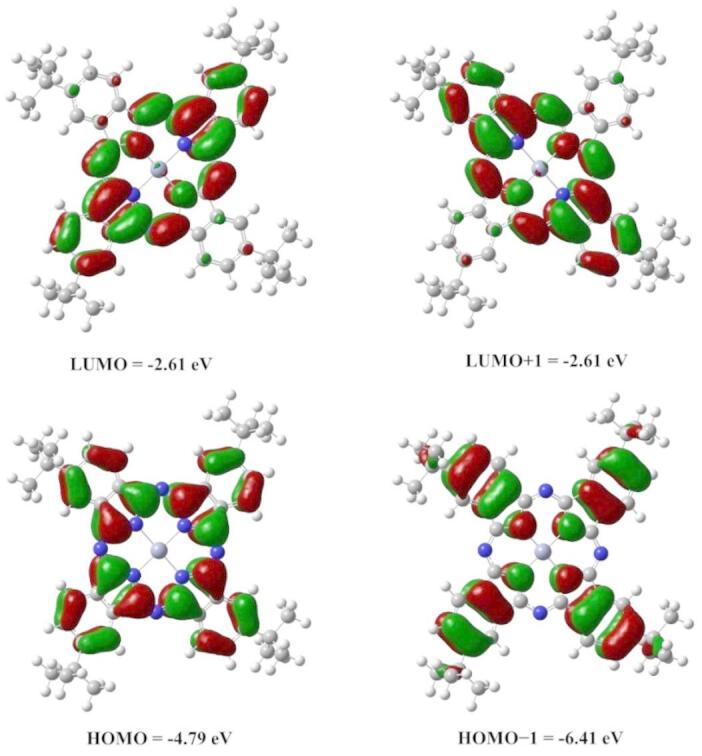
Contour plots of HOMO, HOMO-1, LUMO and LUMO-1 orbitals of ZnTTBPc molecule.

Understanding a molecule’s electronic properties, such as its reactivity, conductivity, and optical qualities, depends on its frontier molecular orbitals (FMOs). FMOs of ZnTTBPc are computed using B3LYP/6-31G(d, p) level of theory, The HOMO-1, HOMO, LUMO, and LUMO + 1 plots for ZnTTBPc molecule are shown in Fig. 2. The ZnTTBPc HOMO is composed of π orbitals that are entirely localized on the Pc framework, with Zn atoms contributing little since they are not engaged in the aromatic π-system, these orbitals are dispersed evenly throughout four isoindole rings. Electron density in the case of LUMO is mostly concentrated on two opposing isoindole rings that are coordinated to the Zn atom. Table 2 reports estimated electronic structure characteristics of H_2_Pc, ZnPc, and ZnPc derivatives^34–38^. Electronic characteristics are strongly influenced by the kind and location of substituents (e.g., alkyl, hydroxyl, carboxylic); their impact on the HOMO and LUMO for ZnPc is noteworthy. Adding or removing electrons from substituents alters the electronic characteristics of phthalocyanine dyes, therefore influencing the distribution of the LUMO and HOMO in space. For example, the LUMO may concentrate on certain areas of the molecule depending on the kind of these substituents whereas the HOMO is prone to delocalization. In ZnTTBPc, substituting tert-butyl groups on ZnPc usually results in a redshift of the Q-band, increased solubility, lower aggregation, and more intense absorption peaks, so improving its fit for optical and electronic uses.

**Table 2:** The LUMO, HOMO and HOMO-LUMO energy levels for H_2_Pc, ZnPc and ZnPc derivatives.

Compd.	HOMO [eV]	LUMO [eV]	HOMO-LUMO energy gap (eV)	Refs.
**H**_**2**_ **Pc**	-5.45	-3.17	2.37	^34^
**ZnPc**	-5.43	-3.10	2.33	^34^
**ZnPc-1**	-5.267	-3.892	1.375	^35^
**ZnPc-2**	-5.187	-3.610	1.577	^35^
**ZnPc1**	-5.38	-3.19	2.19	^36^
**ZnPc2**	-5.55	-3.42	2.13	^36^
**ZnPcB-Ph**	-5.38	-3.43	1.95	^37^
**ZnPcB-N(CH3)**_**2**_	-4.71	-3.18	1.53	^37^
**β-SF**_**5**_ **-ZnPc**	-5.96	-3.77	2.19	^38^
**α-SF**_**5**_ **-ZnPc**	-5.76	-3.51	2.22	^38^
**ZnTTBPc**	-4.79	-2.61	2.18	current work

#### Mulliken charge distribution

The partial atomic charges are estimated using Mulliken charges, which are obtained using the Mulliken population analysis. On the B3LYP/6-31G (d, p) level, the Mulliken charge distribution of a geometry-optimized ZnTTBPc molecule in ground state is computed. Table 3 displays the results. The four coordinated N atoms have the most negative Mulliken charges (-0.721), whereas the center Zn atom has the highest positive Mulliken charges (0.944). In other words, the Zn atom received charge donation from the four N atoms consistent with ligand-to-metal charge transfer in Metallo-phthalocyanines (MPcs)^39–41^. The Zn-atom’s Mulliken charge is less than its formal charge, which is given as + 2, suggesting that the Zn-N bond has both covalent and ionic components^42^. In addition, all of the H atoms in the phthalocyanine nucleus are positively charged, whereas parts of the C atoms are negatively charged.

**Table 3:** Mulliken charges distribution of ZnTTBPc molecule.

Atom	Charge	Atom	Charge
Zn1	0.944	C42, C43, C44, C45	-0.03585
N2, N3, N4, N5	-0.72128	C46, C47, C49, C51, C52, C54, C56, C57	-0.29528
N6, N7, N8, N9	-0.56269	C 48, C50, C53, C55,	-0.3074
C10, C11, C12, C13	0.48685	H58, H59, H60, H61	0.10135
C14, C15, C16, C17	0.48124	H62, H63, H64, H65	0.10383
C18, C19, C20, C21	0.04637	H66, H67, H68, H69	0.08282
C22, C23, C24, C25	0.05208	H70, H73, H80, H86, H89, H95, H101, H103	0.09904
C26, C27, C28, C29	-0.17345	H71, H74, H79, H87, H90, H96, H102, H105	0.10701
C30, C31, C32, C33	-0.13341	H72, H75, H81, H85, H88, H94, H100, H104	0.0981
C34, C35, C36, C37	0.14342	H76, H77, H82, H84, H91, H93, H97, H98	0.10585
C38, C39, C40, C41	-0.12553	H78, H83, H92, H99	0.09619

#### Molecular electrostatic potential (MEP)

**Fig. 3:**
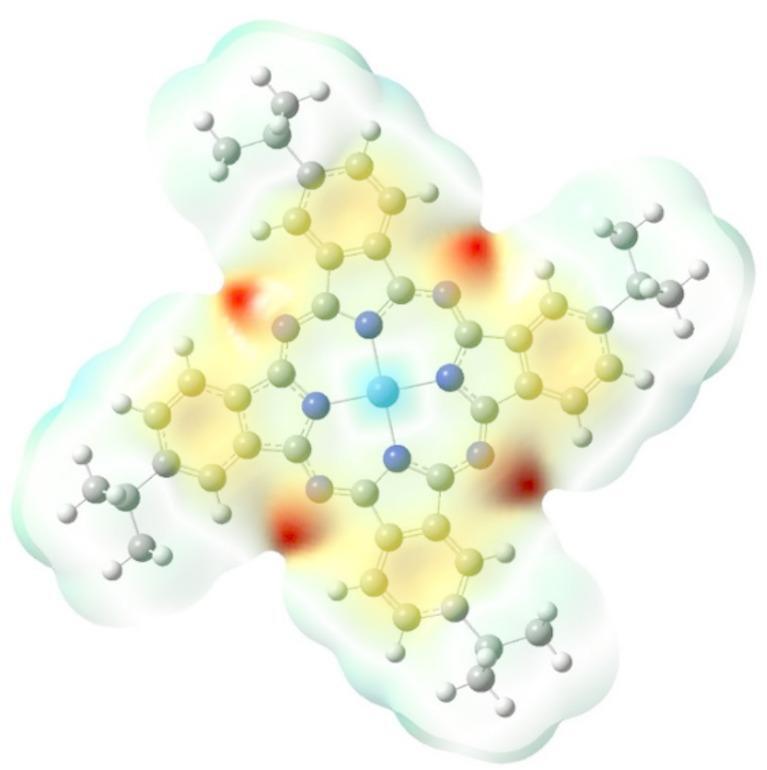
Molecular electrostatic potential for ZnTTBPc molecule.

The molecular electrostatic potential (MEP) is a three-dimensional representation of a molecule’s electrostatic potential. The MEP is a valuable tool for understanding chemical reactivity. It is utilized in research for the creation of novel catalysts, drug-protein interactions, and the design of new materials. The molecular electrostatic potentials (MEPs) of ZnTTBPc are calculated at the B3LYP/6-31G(d, p) level to better understand intermolecular interactions (such as nucleophilic and electrophilic attacks), as displayed in Fig. 3. The different colors on the surface correspond to specific electrostatic potential values, where the red and blue regions of the MEP indicate electrophilic (rich in electrons) and nucleophilic (poor in electrons) activity, respectively. Consequently, different MEP values are represented by various colors, which are arranged in ascending order as red, orange, yellow, green, and blue. The area with the highest negative potential (dark red) is found around the nitrogen atoms of the ZnTTBPc molecule and is excellent for electrophilic interaction. The hydrogen atom has the highest positive charge (blue). The aromatic ring area has a practically neutral potential, which is largely represented in the yellow-green color.

#### Polarizability and hyperpolarizability

Polarizability and hyperpolarizability are significant nonlinear optical (NLO) characteristics that find utility in diverse applications such as second-order harmonic generation (SHG), optical parametric oscillation (OPO), and electro-optic modulation (EOM). The NLO properties such as the mean molecular isotropic polarizability ($\:{\alpha\:}_{p}$), the anisotropy of polarizability ($\:{\varDelta\:\alpha\:}_{p}$) and the total first-order hyperpolarizability ($\:{\beta\:}_{tot}$) of ZnTTBPc were computed at the CAM-B3LYP/6-31G(d, p) level of theory with the help of the theoretical equations described in references^30,43^. As summarized in Table 4, the calculated mean isotropic polarizability is $\:{\alpha\:}_{p}$= 123.2 × 10^− 24^ esu, while the total first-order hyperpolarizability is β_tot_ = 57.97 × 10^− 36^ esu. These values are indicative of pronounced nonlinear optical behavior, suggesting that ZnTTBPc exhibits strong NLO characteristics.

For benchmarking, urea is often employed as a reference NLO material^44^, with values of α_p_ ≈ 3.768 × 10^− 24^ esu and β_tot_ ≈ 0.27 × 10^− 30^ esu calculated at the CAM-B3LYP/6-31G(d, p) level of theory. Relative to urea, ZnTTBPc exhibits a markedly higher isotropic polarizability (about 32-fold larger), indicating strong field-induced polarization. However, its calculated first-order hyperpolarizability is much smaller (only ~ 0.0215% of that of urea). The higher α of ZnTTBPc reflects its extended π-system, while low β is expected for centrosymmetric structures (vs. non-centrosymmetric urea), suggesting that ZnTTBPc has limited second-order nonlinear response despite its pronounced polarizability. i.e. ZnTTBPc may be more promising for applications where high polarizability is advantageous (e.g., third-order NLO processes), rather than for second-order effects such as SHG.

**Table 4:** Isotropic polarizabilities (α_p_) and hyperpolarizability (β) of ZnTTBPc molecule.

	SI (a.u)	esu × 10^− 24^		SI (a.u)	esu× 10^− 36^
**α**_**xx**_	1106.473665	163.97939715	**β**_**xxx**_	-0.0014546	-12.56673
**α**_**xy**_	0.0000052	0.00000077	**β**_**xxy**_	0.0014419	12.45701
**α**_**yy**_	1106.4736772	163.97939896	**β**_**xyy**_	0.0061969	53.53688
**α**_**xz**_	0.0007207	0.00010681	**β**_**yyy**_	0.002108	18.21164
**α**_**yz**_	-0.0002426	-0.00003595	**β**_**xxz**_	0.0018892	16.32137
**α**_**zz**_	281.8818179	41.77488541	**β**_**xyz**_	0.0000383	0.33089
**α**_**p**_	831.60972003	**123.24456051**	**β**_**yyz**_	0.0027517	23.77276
**Δα**_**p**_	824.5918532	122.20451264	**β**_**xzz**_	0.0000405	0.34989
			**β**_**yzz**_	-0.0000019	-0.01641
			**β**_**zzz**_	-0.0077335	-66.81203
			**β**_**tot**_	0.00671026	**57.97199**

#### Absorption spectra

**Fig. 4:**
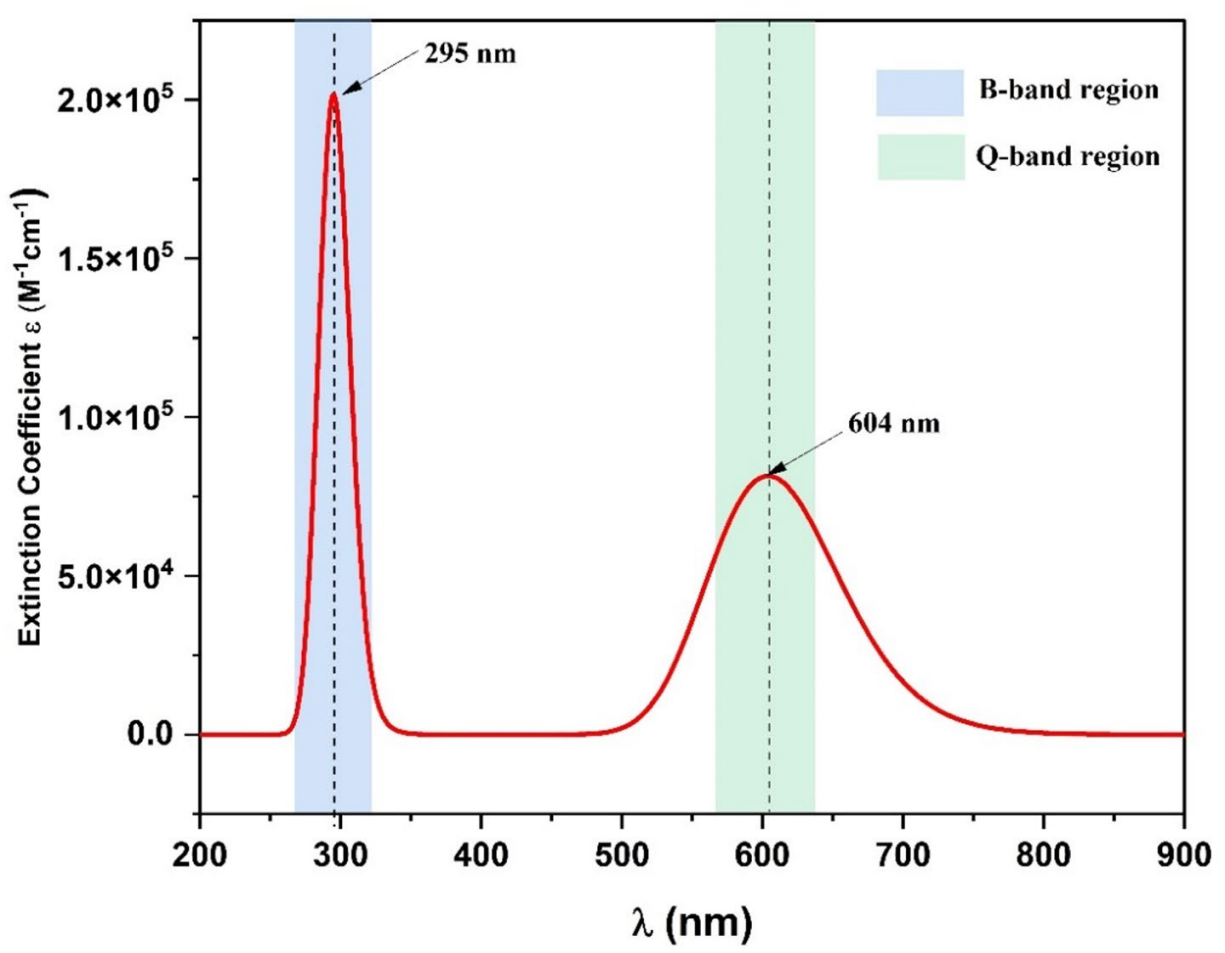
The simulated absorption spectra of ZnTTBPc molecule.

UV-Vis absorption spectra of the ZnTTBPc molecule was calculated by the TD-DFT/ CAM-B3LYP/6-31G(d, p) level of theory in the gas phase by considering the lowest 15 excited states. Figure 4 shows the corresponding UV-Vis absorption spectra. The obtained results of maximum absorption wavelengths (λ_max_), electronic vertical transition energies (E_ver_) from the ground state to an excited state, oscillator strengths (f), and transition characters of ZnTTBPc molecule are listed in Table S5. The simulated spectrum shows two dominant absorption regions: a sharp B-band (Soret band) at 295 nm with a high oscillator strength of 1.13882 and a broader Q-band at 604 nm correspond to the oscillator strength of 0.5621, both arising from π–π* electronic transitions within the conjugated phthalocyanine macrocycle.

### Structural and optical properties of ZnTTBPc thin film

#### Crystal structure of ZnTTBPc thin films

**Fig. 5:**
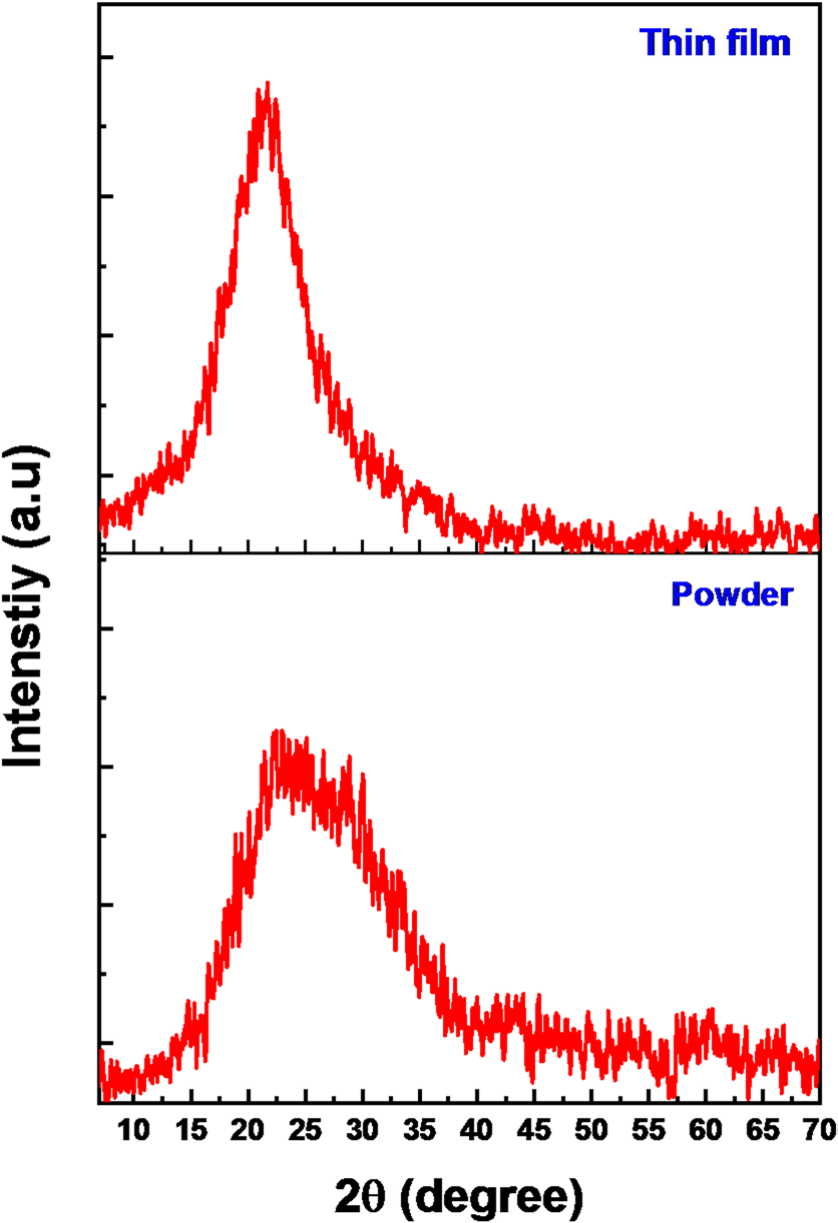
XRD pattern for ZnTTBPc in powder and thin film form.

**Fig. 6:**
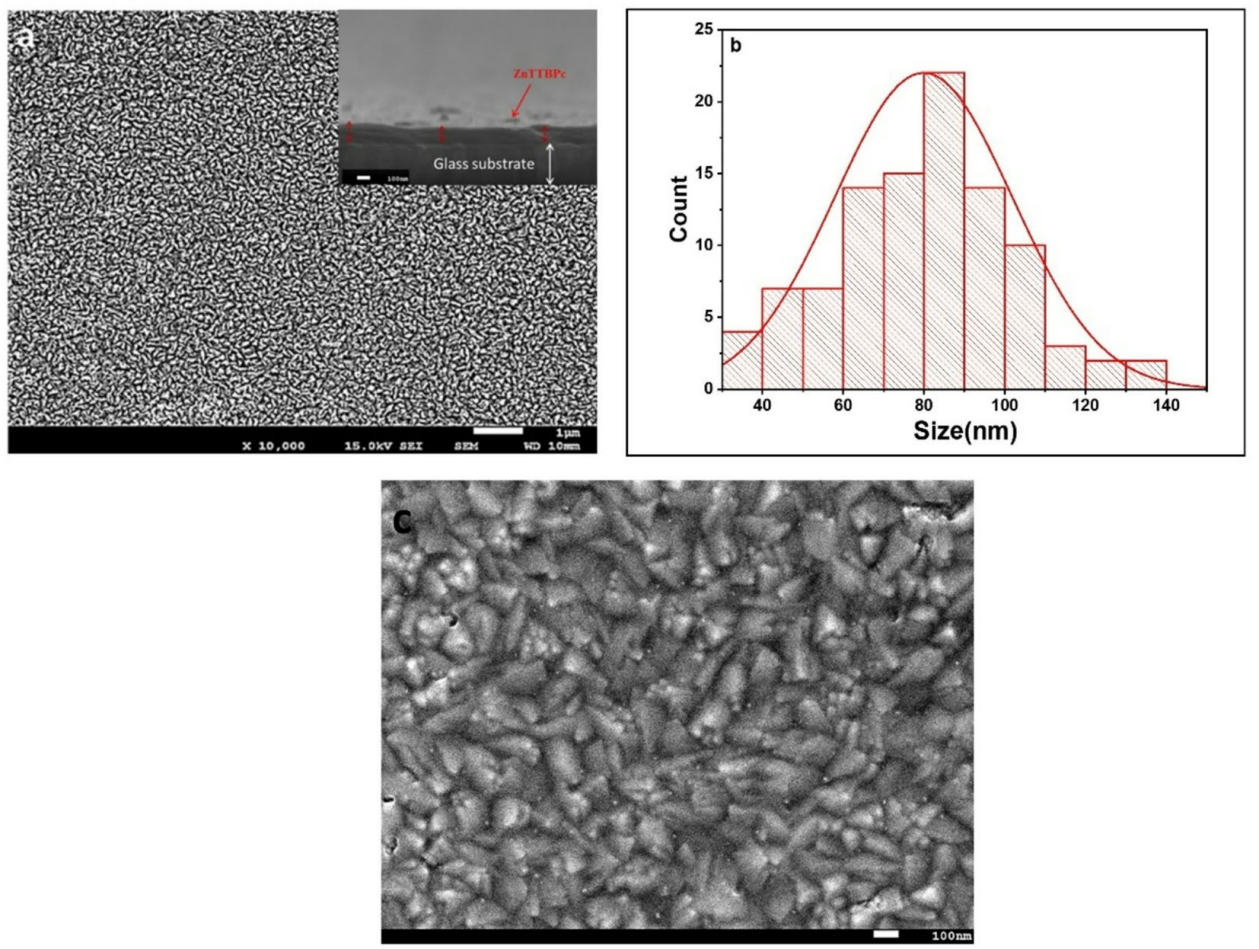
(**a**) Top-view FE-SEM image of as-deposited ZnTTBPc thin film, and the inset shows cross-sectional FE-SEM image, (**b**) Histogram of the grain size distribution, and (**c**) the high magnified FE-SEM image for ZnTTBPc thin film.

Figure 5 illustrates XRD patterns of ZnTTBPc in both powder and as-deposited film states. The XRD patterns indicate that both the ZnTTBPc powder and the as-deposited film exhibit an amorphous structure, as evidenced by the lack of crystalline peaks. FE-SEM is an outstanding technique for studying the surface morphology of thin films. This information can be used to get a better understanding of microstructure and can also be used to optimize the deposition of thin films and develop new thin film materials with improved properties and performance. Figure 6(a) displays FE-SEM image and particle distribution of ZnTTBPc thin films. The thin films in their as-deposited condition are homogeneous, with no fractures or pinholes, and efficiently cover the substrate as shown in Fig. 6(a). The image implies that thin films have advantageous properties that make them appropriate for a broad variety of applications. Furthermore, the cross-section view in insert Fig. 6(a) confirms the successful growth of spin-coated ZnTTBPc thin film with a thickness of ~ 100 nm.

A higher-magnification is shown in Fig. 6(b) enables the observation of the internal contrast of a nanoflake, which is regarded as the fundamental form of ZnTTBPc. The grain size distributions of ZnTTBPc are shown in Fig. 6(c). Using Image J software, the average grain sizes of ZnTTBPc thin films formed on quartz substrates are calculated and found to be about 80 ± 3 nm. According to the study, the average grain size values indicate that the generated film has a nanostructured property.

**Fig. 7:**
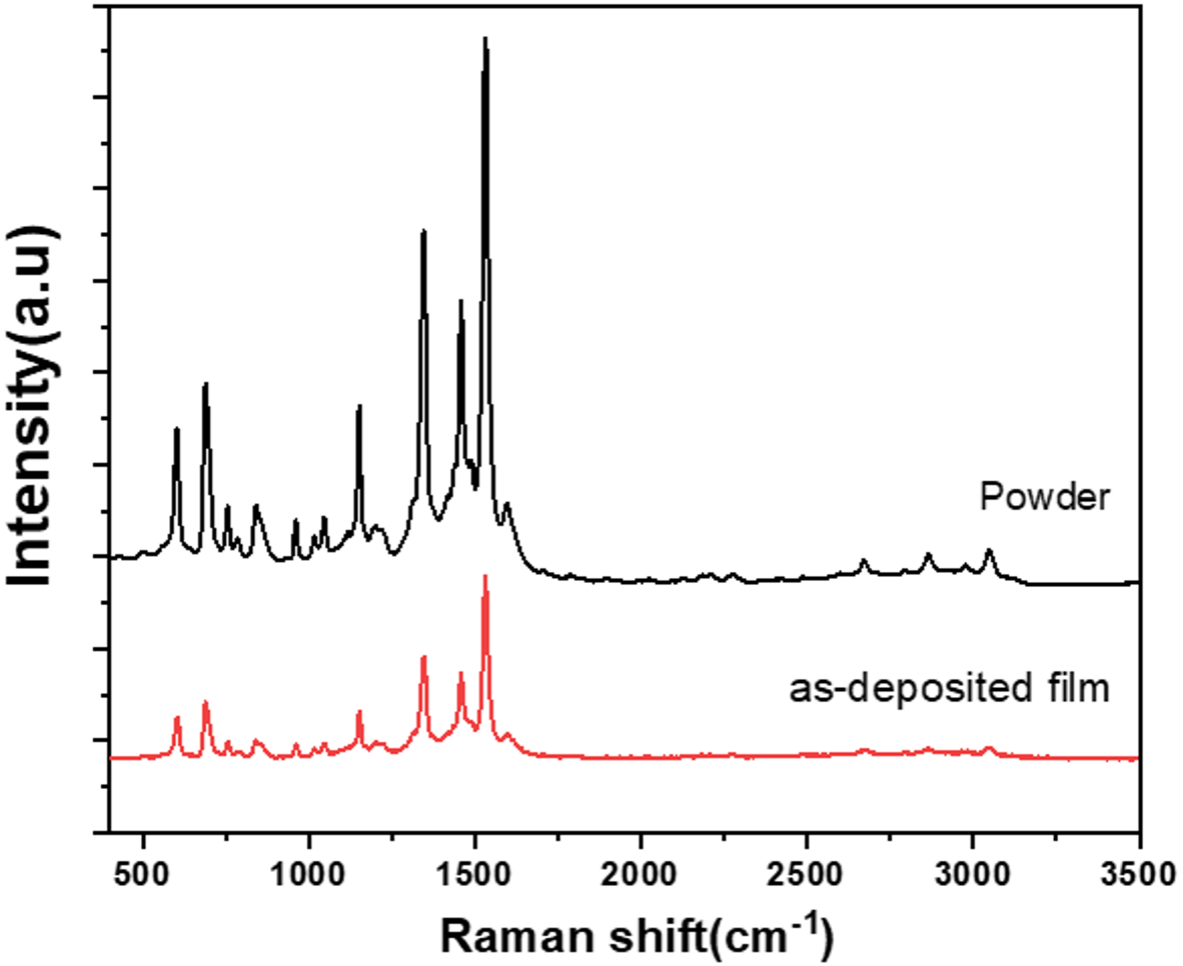
Raman spectra for ZnTTBPc powder and investigated thin film.

Figure 7 illustrates the Raman spectra obtained for ZnTTBPc powder and the as-deposited thin film. The most intense spectral peak is seen at a wavenumber of 1530 cm^− 1^, indicating a significant association between a metal ion and a phthalocyanine molecule^3^. ZnTTBPc powder and as deposited thin film exhibits display a significant displacement of the prominent Raman peaks at 1346 ± 3 and 1531 ± 3 cm^− 1^, as compared to the corresponding peaks seen in ZnPc powders and thin films, which are measured at 1337.59 and 1508.14 cm^− 1^, respectively^45–47^.These findings are the result of a structural alteration in ZnTTBPc caused by the tert-butyl group acting as a substituted group. Moreover, a strong consensus exists on the similarity seen in the Raman spectra of ZnTTBPc in powdered form and those of ZnTTBPc thin films. Thus, spin-coating appears to be a potential approach for producing high-quality ZnTTBPc thin films.

#### Optical properties of ZnTTBPc thin film

**Fig. 8:**
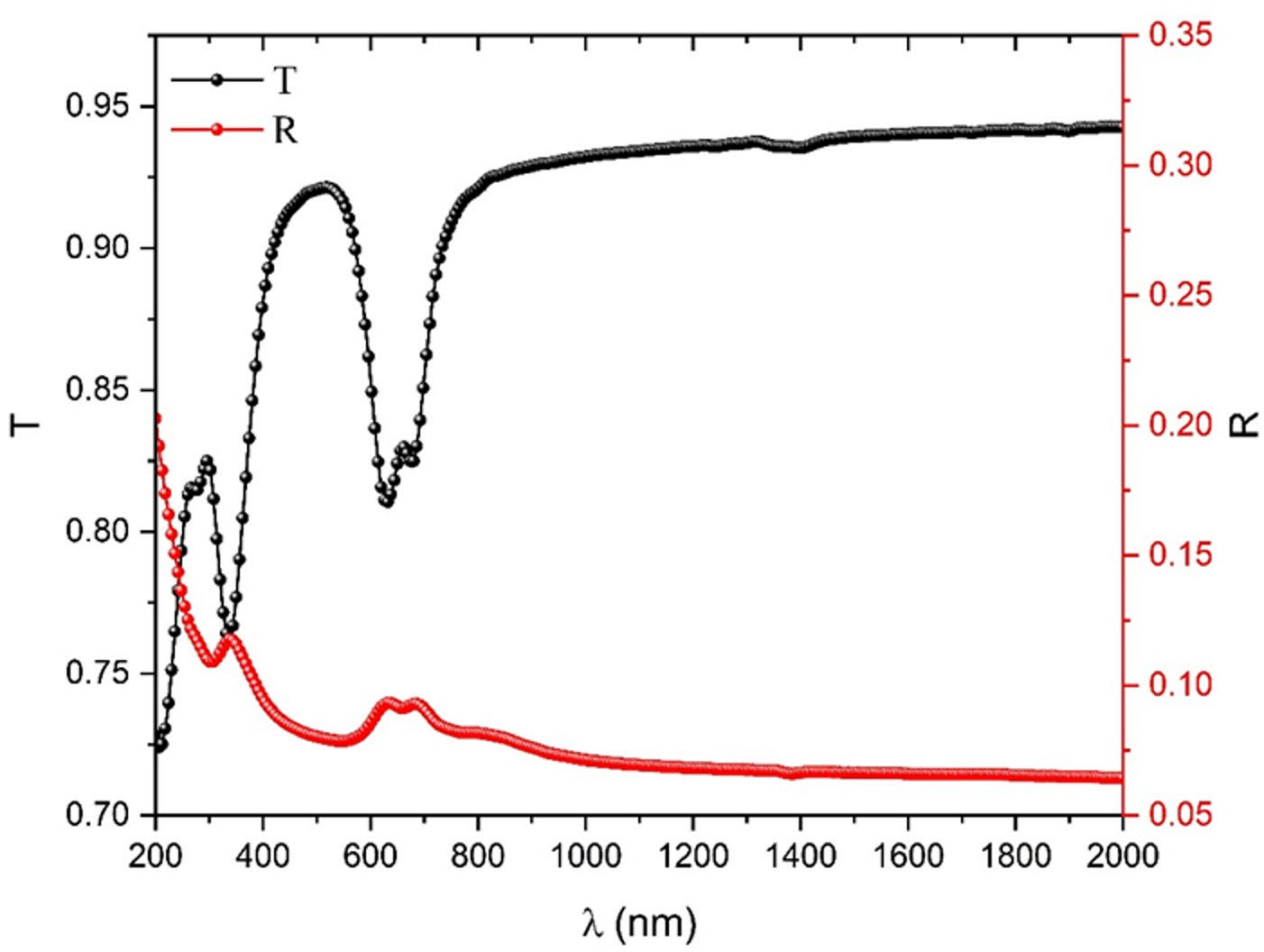
The optical transmission T(λ) and reflection R(λ) for as-deposited ZnTTBPc thin film.

**Fig. 9:**
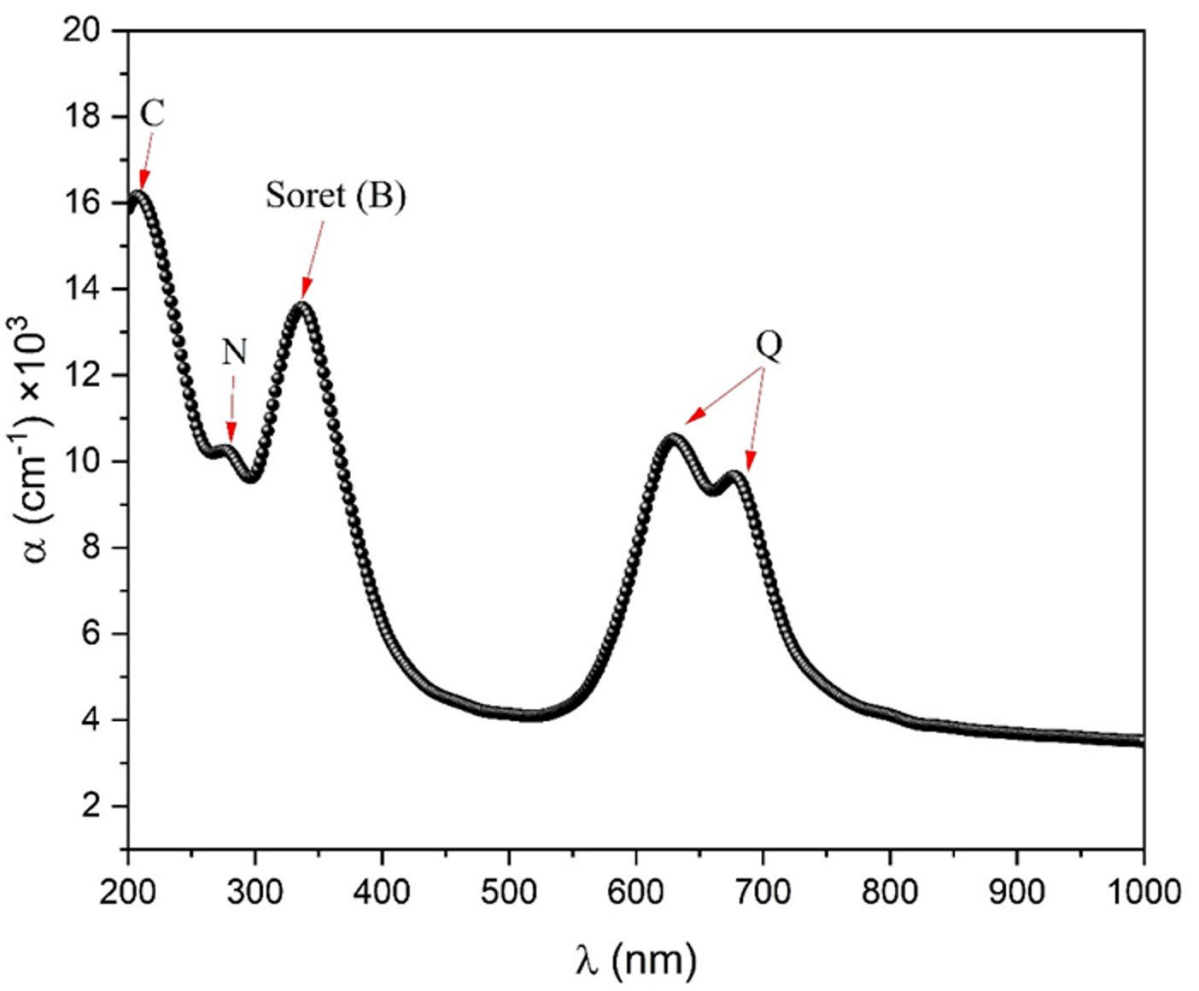
Spectral distribution of absorption coefficient for ZnTTBPc thin film.

Figure 8 illustrates spectral distribution of T (λ) and R (λ) for the as-deposited ZnTTBPc thin film within the wavelength range of 200 to 2000 nm. At shorter wavelengths, specifically (λ < 800 nm), the combined values of R (λ) and T (λ) indicate a total that is less than unity, implying the occurrence of absorption. At wavelengths greater than 800 nm, the combined values of R (λ) and T (λ) go towards unity, indicating the presence of an optically transparent region with no absorption of light. In addition, the observable range of the T (λ) spectrum, a substantial and extensive absorption band is present, suggesting the potential utility of ZnTTBPc material in the photovoltaic applications. The absorption coefficient (α) describes the ability of a material to absorb incoming photon energy. This information pertains to the electronic transition inside the material. The following formula is used to compute the absorption coefficient^48^:

1 $$\:{\upalpha\:}=\frac{1}{\text{d}}\text{ln}\left(\frac{1}{\text{T}}\right)$$

where, d is the film thickness. The UV-visible spectrum of phthalocyanines arises from the presence of molecular orbitals inside the aromatic 18 π-electron system, as well as the overlapping of orbitals on the central metal atom^3^. Figure 9 displays the absorption coefficient spectra for the ZnTTBPc thin film in its as-deposited state. The spectra show the Q and B (Soret) bands, which are all typical of MPcs^2,3,35^ at wavelengths ranging from 550 to 700 nm and 300 to 400 nm, respectively. In addition, the N and C bands are visible in the ultraviolet spectrum, due to the filled Zn^+ 2^ d^10^ electronic configurations. The B band has a single peak at 337 nm and exists due to the direct electrical transition of the Pc ring’s (π) to its (π*). The Q band (Q_x_ and Q_y_) has two distinct peaks at 628 and 676 nm. The energy discrepancy between the two peaks in the doublet is approximately 0.14 eV, with the higher energy peak (Q_x_) exhibiting a greater intensity compared to the lower energy peak (Q_y_). This behavior is characteristic of the α-phase of ZnTTBPc^20^.

**Fig. 10:**
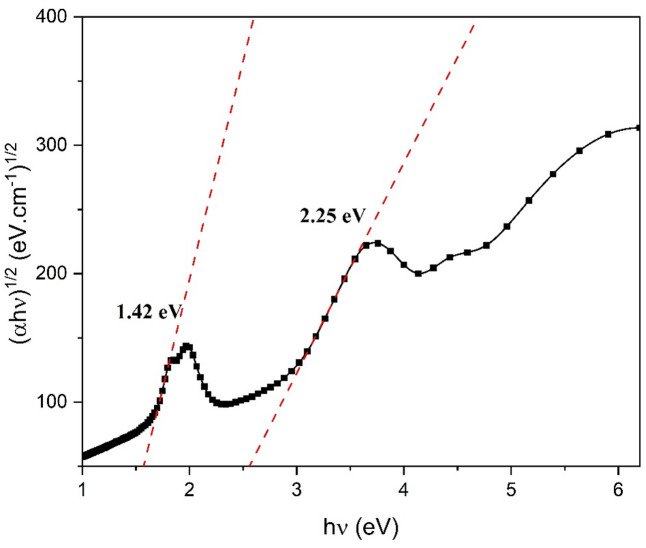
(ahυ)^1/2^ versus photon energy (hυ) for ZnTTBPc thin film.

Tauc’s relation is an extremely useful tool for studying the optical characteristics of materials, including organic thin films. It is a semi-empirical formula describing the interband absorption coefficient as a function of photon energy in the absorption edge region (10^4^ cm^− 1^). The following equation gives it ^49,50^:

2 $$\:\alpha\:h\nu\:=A{(h\nu\:-{E}_{g}^{opt})}^{r}$$

where, hv is photon energy, A is the constant,$\:\:{E}_{g}^{opt}$ is the energy of the optical band gap, and r controls the kind of optical transition. The experimental data is found to exhibit the highest level of agreement with Eq. (2) when *r* = 2, suggesting that ZnTTBPc has an indirect band gap. The plot in Fig. 10 illustrates the relationship between ($\:\alpha\:h\nu\:$) ^1/2^ and $\:h\nu\:$ for the film in its as-deposited state. Table 5 presents the estimated values of the $\:{E}_{g}^{Q}$ and $\:{E}_{g}^{B}$ or each electronic transition, which are obtained by projecting the linear sections of the curves onto the (αhν) ^2^ = 0 axis. The $\:\:{\text{E}}_{\text{g}}^{\text{Q}}$ and $\:{\text{E}}_{\text{g}}^{\text{B}}$ for ZnTTBPc is smaller than $\:{\text{E}}_{\text{g}}^{\text{Q}}$ and $\:{\text{E}}_{\text{g}}^{\text{B}}$ for spin coated ZnPcBzF^51^ and (4TFEO) 4-ZnPc^3^. The disparity in $\:{E}_{g}^{opt}$can be ascribed to the tert-butyl groups in ZnTTBPc, which significantly affect electronic characteristics. The tert-butyl groups in ZnTTBPc are very significant for varying their electronic properties because they change how the molecules are packed together and how the electrons are spread out. Their bulky structure prevents close molecular stacking, reducing intermolecular interactions and, therefore, reducing the energy band gap^52^.

##### The dispersion analysis

**Fig. 11:**
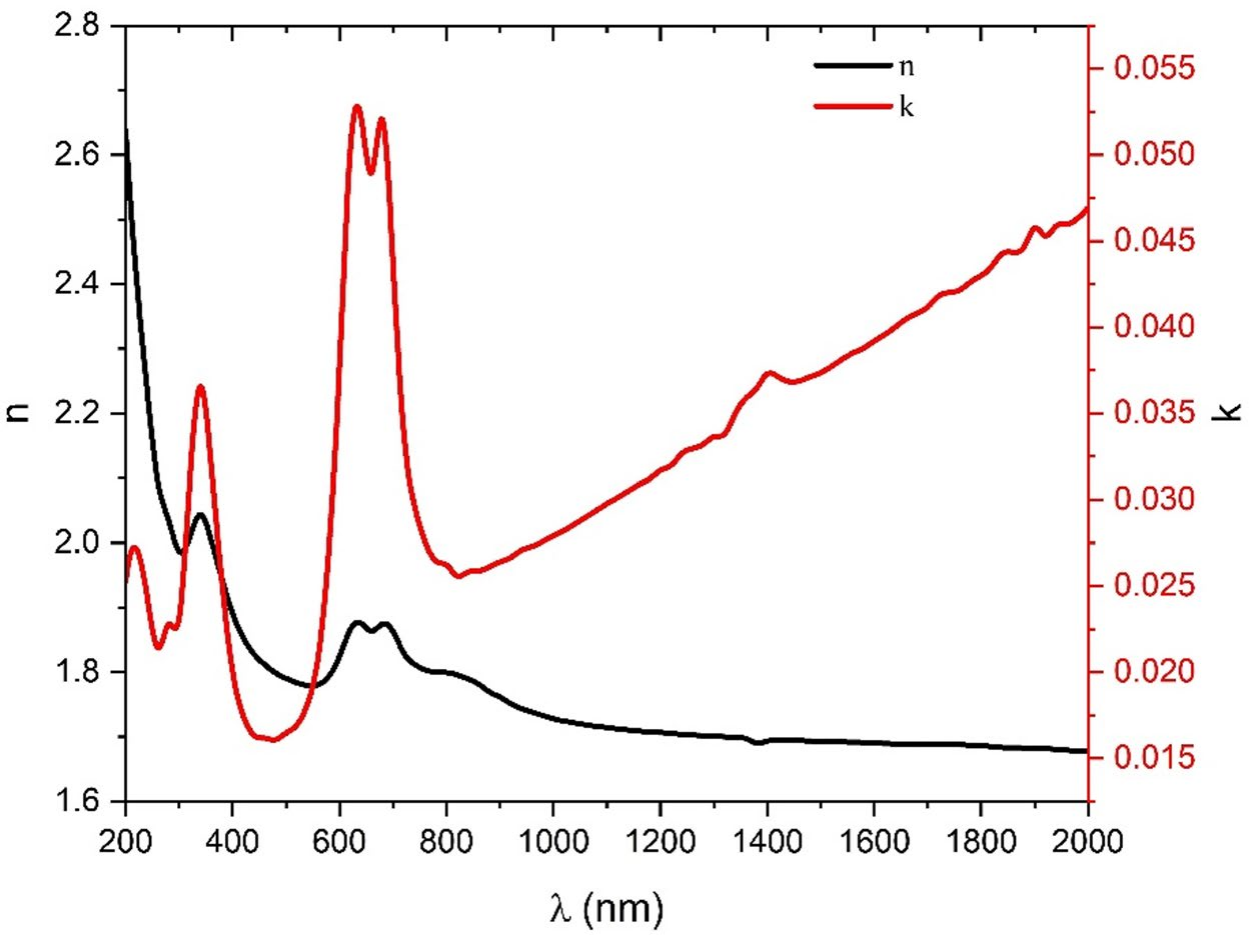
Refractive index and Extinction coefficient of ZnTTBPc thin film as a function of wavelength.

The optical constants of the thin film under consideration, such as the refractive index n and the extinction coefficient k, are calculated using the formulae below^53,54^:

3 $$\:\left\{ {\begin{array}{*{20}c} \begin{gathered} k = \frac{{\alpha \:\lambda \:}}{{4\pi \:}}\:\:\: \hfill \\ n = \frac{{1 + R}}{{1 - R}} + \sqrt {\frac{{4R}}{{(1 - R)^{2} }} - k^{2} } \hfill \\ \end{gathered} \\ \end{array} } \right\}$$

n* = n - jk denotes the complex refractive index. The imaginary component (extinction coefficient, k) represents the amount of optical loss per unit distance in the medium. Figure 11 depicts the variation of the k for ZnTTBPc films with wavelength. It illustrates that at a wavelength about 800 nm, the extinction coefficient k starts to increase with increasing wavelength, indicating the existence of free carriers in ZnTTBPc films^55–57^. While the refractive index is velocity-dependent, the real component of the complex **n**, carries information regarding electronic polarization^58^. Figure 11 displays the **n**, of the investigated ZnTTBPc film as a function of light wavelength. The **n**, exhibits typical dispersion at wavelengths greater than 800 nm, indicating that it decreases as the wavelength increases. Nevertheless, as the wavelength increases, the **n** becomes more stable, suggesting that the films lose their dispersive properties.

**Fig. 12:**
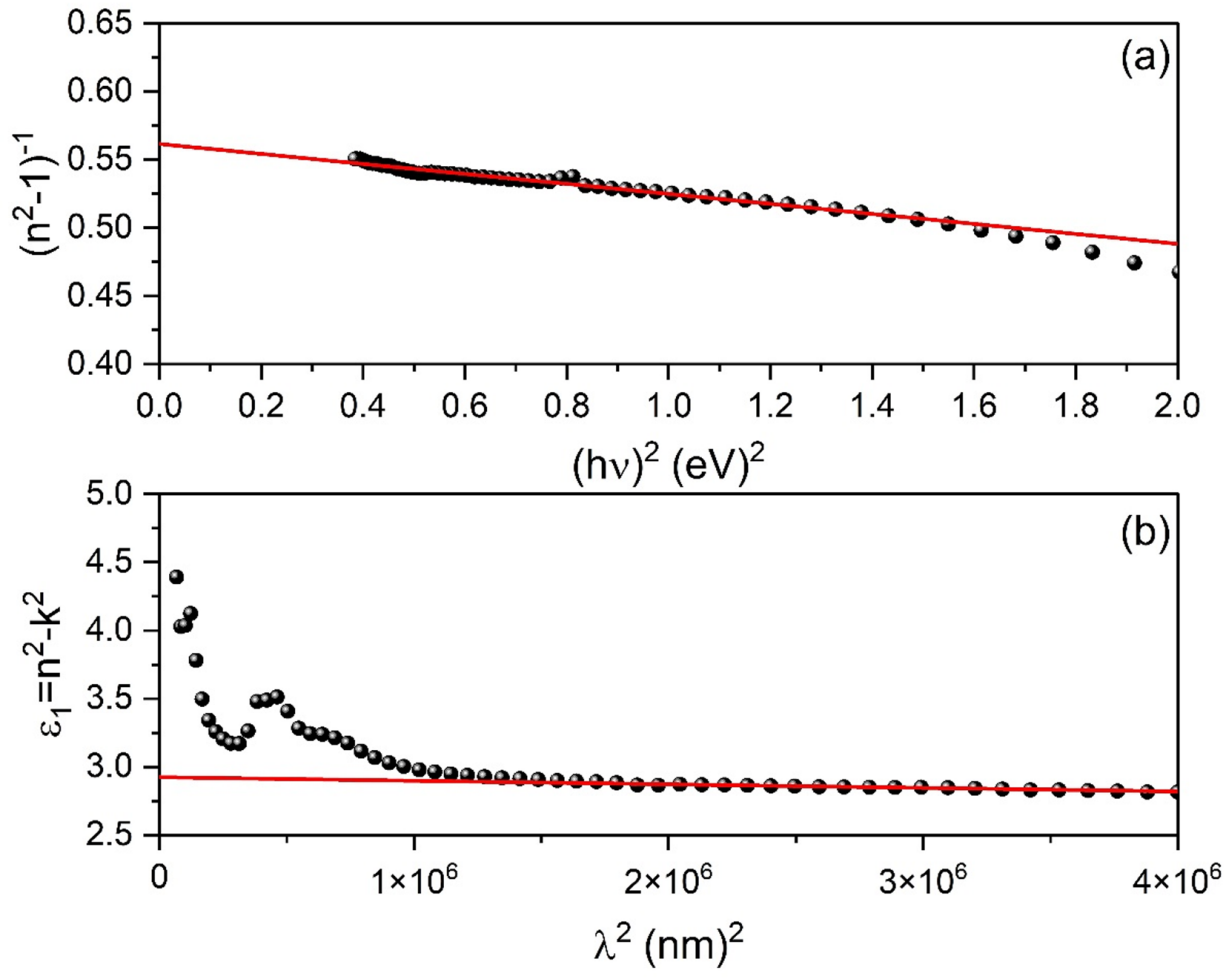
(**a**) Plots of (n^2^ − 1)^−1^ vs. (hν)^2^ and (**b**) Plots of ε_1_ vs. (λ)^2^ for ZnTTBPc thin film.

The dispersion of the as-deposited thin film’s refractive index is investigated using a single oscillator model, which is represented by the Wemple and DiDomenico (WDD) relationship^59^:

4 $$\:{n}^{2}=1+\frac{{E}_{d}{E}_{o}}{{E}_{o}^{2}-{\left(h\nu\:\right)}^{2}}$$

where, $\:{E}_{d}$ denotes the dispersion energy, hv the photon energy, and $\:{E}_{o}$ the single-oscillator energy. $\:{E}_{d}$ and $\:{E}_{o}$ dispersion parameters may be readily derived by graphing $\:{({n}^{2}-1)}^{-1}$ against $\:{\left(h\upsilon\:\right)}^{2}$as illustrated in Fig. 12(a). The slope $\:{\left({E}_{o}{E}_{d}\right)}^{-1}$ and the intercept $\:\left({E}_{o}/{E}_{d}\right)$ on the vertical axis may be used to directly calculate the values of E_d_ and E_o_. Table 5 shows the values obtained for the dispersion parameters $\:{E}_{o}$ and $\:{E}_{d}$ for the ZnTTBPc thin film. To a decent approximation, $\:{E}_{o}$ is an average energy gap that scales with the optical band gap$\:\:{E}_{g}^{opt}$^60^ ( $\:{E}_{o}\approx\:2{E}_{g}^{Q}$). The lower values of values of E_d_ and E_o_ for ZnTTBPc thin film compared to (4TFEO) 4-ZnPc thin film^3^ are attributable to the presence of tert-butyl groups in ZnTTBPc. These groups influence molecular arrangement. This decreases correlates with the overall lowering of $\:{E}_{g}^{opt}$ in the ZnTTBPc thin film.

##### High frequency lattice dielectric constant and charge carrier concentration

According to the Drude theory, the real part of the dielectric constant is given by^61^:

5 $$\:{\epsilon\:}_{1}\left(\omega\:\right)={\epsilon\:}_{\infty\:}-\frac{{\omega\:}_{p}^{2}}{{\omega\:}^{2}+{\gamma\:}^{2}}\approx\:{\epsilon\:}_{\infty\:}-\frac{{\omega\:}_{p}^{2}}{{\omega\:}^{2}}$$

where, ε_∞_ is the high frequency lattice dielectric constant, ω is the angular frequency$\:\:(\omega\:=2\pi\:c/\lambda\:)$, γ is the damping rate, and ω_P_ is the plasma frequency given by $\:{\omega\:}_{p}={(N{e}^{2}/{\epsilon\:}_{o}{m}^{*})}^{1/2}$. The last equation can be written as follows:

6 $$\:{\epsilon\:}_{1}={\epsilon\:}_{\infty\:}-\left(\frac{{e}^{2}}{4\:{\pi\:}^{2}\:{c}^{2}{\epsilon\:}_{o}}\right)\left(\frac{N}{{m}^{*}}\right){\lambda\:}^{2}$$

where, $\:N/{m}^{*}$ is the charge carrier concentration to effective mass ratio, e is the electronic charge, and $\:{\epsilon\:}_{o}$ is the vacuum permittivity. Figure 12(b) depicts the fluctuation of $\:{\epsilon\:}_{1}$ vs. $\:{\lambda\:}^{2}$ for ZnTTBPc film. The dependency of $\:{\epsilon\:}_{1}$ on $\:{\lambda\:}^{2}$ is linear at longer wavelengths, which may be extended to zero wavelength (λ^2^ = 0) to compute the lattice dielectric constant $\:{\epsilon\:}_{\infty\:}$, and the slope of this linear component can be used to derive the value of$\:\:N/{m}^{*}$. The calculated values of $\:{\epsilon\:}_{\infty\:}$ and $\:\:N/{m}^{*}\:$ are listed in Table 5.

**Table 5:** Estimated optical parameters of ZnTTBPc thin film.

Optical parameters	
$\:{\varvec{E}}_{\varvec{g}}^{\varvec{Q}}$ **(eV)**	1.42
$\:{\varvec{E}}_{\varvec{g}}^{\varvec{B}}$ **(eV)**	2.25
**E**_**d**_ **(eV)**	6.97
**E**_**o**_ **(eV)**	3.91
**N∕m** ^*^	3.195 × 10^55^
**ε**_**∞**_	2.92

##### Dielectric characterization

The complex dielectric constant is an essential material characteristic for optoelectronic applications. The real and imaginary components of the dielectric constant provide insights into the degree of light deceleration and energy absorption from the electric field inside the material^53^. The real $\:{\epsilon\:}_{1}$ and imaginary $\:{\epsilon\:}_{2}$ components of the dielectric constant can be calculated from n and k^53,54^:

7 $$\:{\epsilon\:}_{1}\left(\omega\:\right)={n}^{2}-{k}^{2}\:\:and\:\:\:\:{\epsilon\:}_{2}\left(\omega\:\right)=2\text{n}\text{k}\:$$

**Fig. 13:**
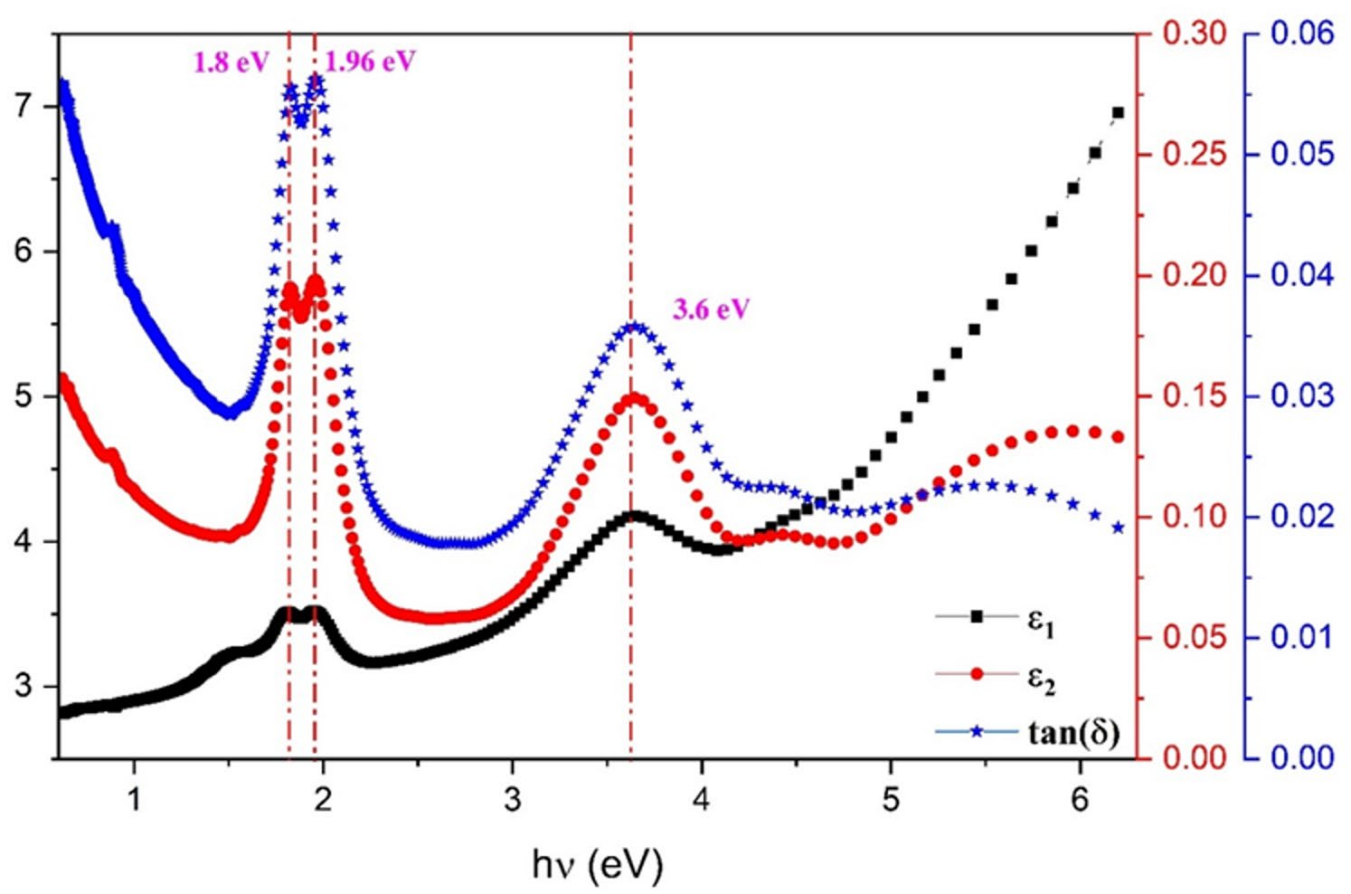
The variation of ε_1_, ε_2_ and tan δ with hν for ZnTTBPc thin film.

The real portion $\:{\epsilon\:}_{1}$ represents dispersion, whereas the imaginary part $\:{\epsilon\:}_{2}$ represents the wave’s dissipative rate in the medium^62^. Figure 13 depicts the fluctuation of the dielectric constant components $\:{\epsilon\:}_{1}$ and $\:{\epsilon\:}_{2}$ with photon energy hν of the examined ZnTTBPc thin film. As seen in the graph, the value of the real component is greater than the value of the imaginary part. Three peaks in the $\:{\epsilon\:}_{2}$ spectra are located at 1.8, 1.96, and 3.6 eV. A dielectric substance’s dissipation factor (loss tangent)$\:\text{tan}\delta\:$, is a measure of how much energy is lost when an alternating electric field is applied to the material. It is a quantity nwith no dimensions, and can be determined using the following equation^63,64^:

8 $$\:\text{tan}\delta\:=\frac{{\epsilon\:}_{2}}{{\epsilon\:}_{1}}$$

The $\:\text{tan}\delta\:$ (see Fig. 13) a significant characteristic of dielectric materials that is taken into account in several applications. In the context of capacitors, it is advantageous to have a low dissipation factor in order to mitigate energy losses. In the context of insulators, it is important to have a low dissipation factor in order to decrease the occurrence of leakage currents. In the context of waveguides, it is advantageous to have a low dissipation factor in order to limit the attenuation of signals.

##### Nonlinear optical properties of ZnTTBPc films

**Fig. 14:**
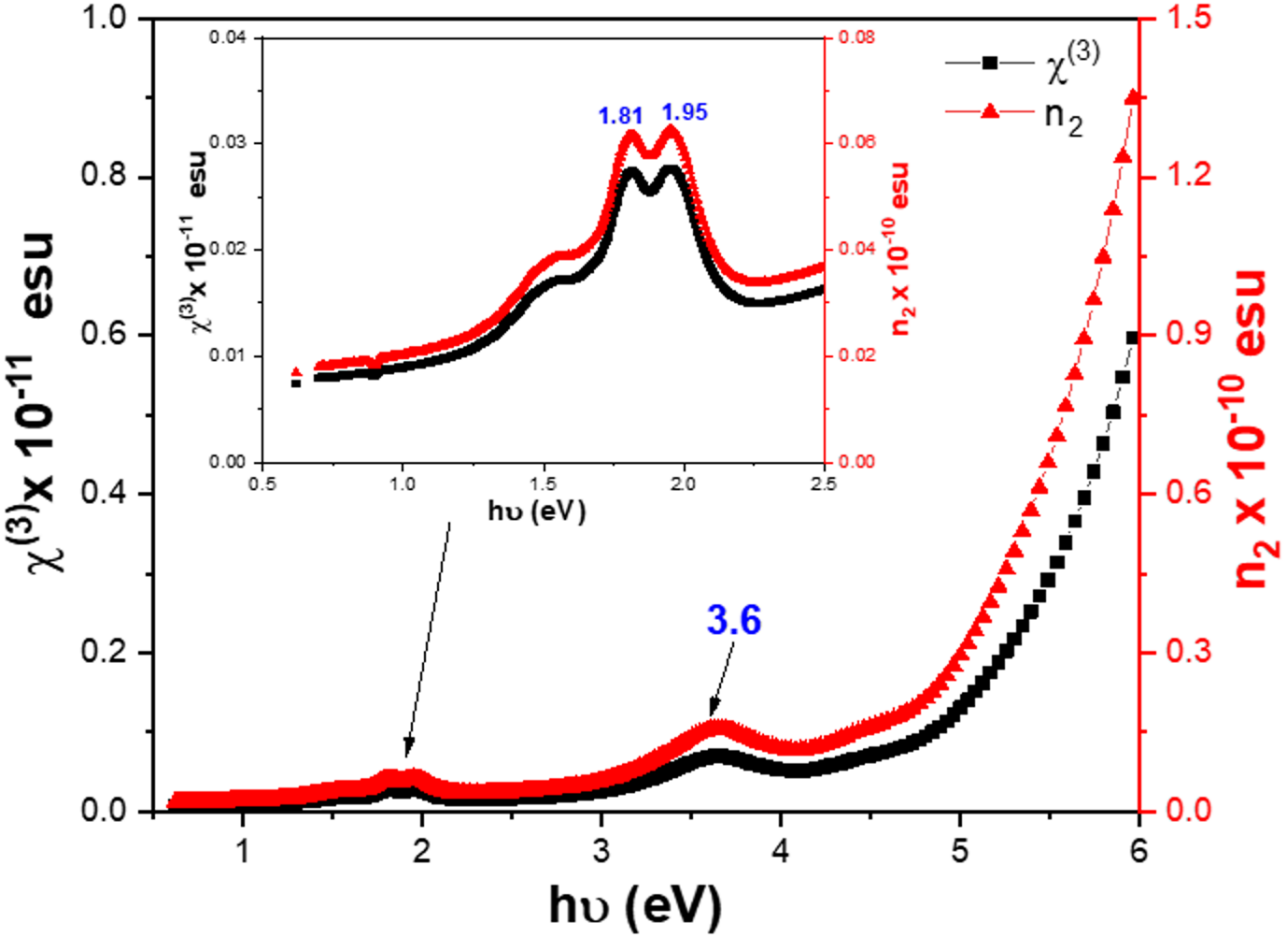
Variation of (**a**) χ ^(3)^ and (**b**) n_2_ vs. hν for ZnTTBPc thin film.

MPcs thin films exhibit notable nonlinear optical (NLO) characteristics because of their higher -electron delocalization. As a consequence, a promising material for nonlinearity devices is an excellent choice for optical limiting applications^3^. According to Miller’s general rule, the calculation of the nonlinear third-linear optical susceptibility $\:{\chi\:}^{\left(3\right)}$ and nonlinear refractive index ($\:{n}_{2}$) can be performed using the formulae provided in references^3,65–68^:

9 $$\:{\chi\:}^{\left(3\right)}=1.7\times\:{10}^{-10}(\frac{{n}^{2}-1}{4\pi\:}{)}^{4}=1.7\times\:{10}^{-10}{\left[\frac{{E}_{o}{E}_{d}}{4\pi\:\left({E}_{o}^{2}-{\left(h\nu\:\right)}^{2}\right)}\right]}^{4}$$

10 $$\:{\:\:\:\:\:\:\:\:\:\:n}_{2}=\frac{12\pi\:}{{n}_{o}}{\chi\:}^{\left(3\right)}\:$$

where, n_o_ denotes the static refractive index. The variations of $\:{\chi\:}^{\left(3\right)}$and $\:{n}_{2}$ for ZnTTBPc thin film versus $\:h\nu\:$ are shown in Fig. 14. The plots of$\:{\:\chi\:}^{\left(3\right)}$and $\:{n}_{2}$ show the viability of optical switching using ZnTTBPc thin films with significant peaks are located at 1.81, 1.95, and 3.6 eV, within the hν range (0.5 to 6 eV). The $\:{\chi\:}^{\left(3\right)}$and $\:{n}_{2}$for ZnTTBPc thin film are on the order of 10^− 11^ and 10^− 10^ esu, respectively. The values of $\:{\chi\:}^{\left(3\right)}$and $\:{n}_{2}$ at the zero-frequency (hυ = 0) were calculated using Eqs. 9 and 10. These calculated values are shown in Table 6 and are compared with those of other related metal MPcs thin films^3,65,69–77^. Analysis of the thin film’s nonlinear optical characteristics reveals that the ZnTTBPc thin film has exceptional capabilities, making its strong nonlinear optical response makes it suitable for a wide range of applications.

Nonlinear absorption coefficient β_c_ is a nonlinear process when two photons are absorbed simultaneously, leading to a direct transition from the ground state to high-energy states. Sheik-Bahae et al. discovered an empirical correlation between the βc coefficient and $\:{\text{E}}_{\text{g}}^{\text{O}\text{p}\text{t}}$ for the following^78–81^:

11 $$\:{\beta\:}_{c}\left(\omega\:\right)=\frac{3100\sqrt{21}{\left[\left(2h\nu\:/{E}_{g}^{Opt}\right)-1\right]}^{3/2}}{{n}^{2}\:{{E}_{g}^{Opt}}^{3}{\left(2h\upsilon\:/{E}_{g}^{Opt}\right)}^{5}}$$

**Fig. 15:**
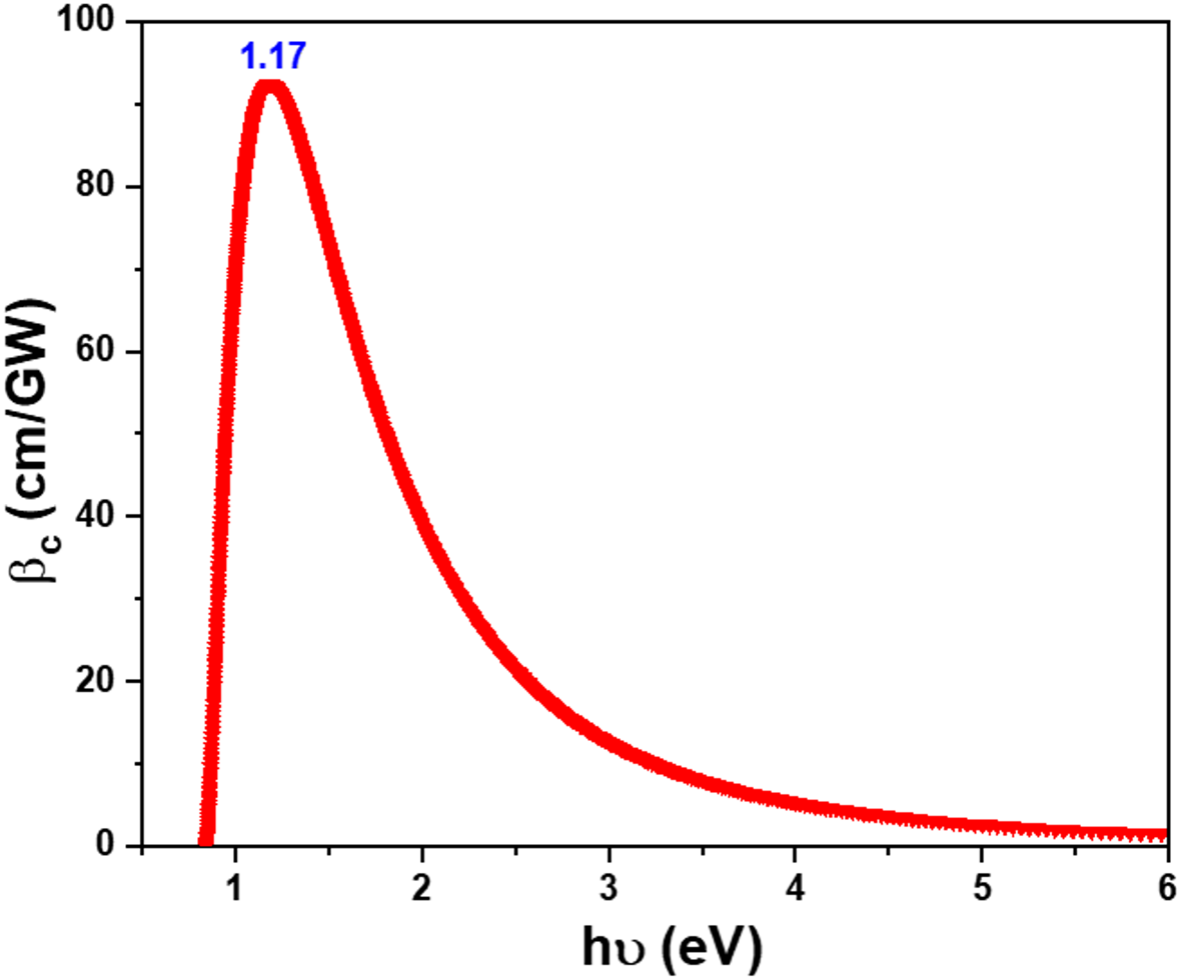
The variation of β_c_, vs. hν for ZnTTBPc thin film.

The β_c_ value grows with the increase in hv up to it reaches its highest value ( $\:{{\varvec{\beta\:}}_{\varvec{c}}}_{\varvec{m}\varvec{a}\varvec{x}}$) and then declines again (see Fig. 15; Table 6). The nonlinear absorption process is resonant, indicating that it is most effective when the photon energy (hv) falls within the range of ($\:{\text{E}}_{\text{g}}^{\text{O}\text{p}\text{t}}$⁄2 < hυ <$\:{\text{E}}_{\text{g}}^{\text{O}\text{p}\text{t}}$). The calculated values of the nonlinear parameters (χ^3^, n_2_, and β_c_) vary owing to the influence of (i) the central metal of phthalocyanines, (ii) the thicknesses, and (iii) the nature of the substrate, as shown in Table 6.

**Table 6:** Comparison of χ ^(3)^, n_2_ and β_c_ for ZnTTBPc thin film with other phthalocyanine compounds.

MPcs /Substrate, Thickness	$\:{\varvec{\upchi\:}\:}^{\left(3\right)}$esu (hυ→0)	$\:{\mathbf{n}}_{2}\:$esu (hυ→0)	$\:{{\varvec{\beta\:}}_{\varvec{c}}}_{\varvec{m}\varvec{a}\varvec{x}}$ ( cm/GW)	Ref.
ZnTTBPc/ Quartz, 100 nm	6.89 × 10^− 14^	1.55 × 10^− 12^	93	Current work
TiPcCl_2_/Quartz, 250 nm	1.35 × 10^− 12^	2.34 × 10^− 11^	166	^69^
TiPcCl_2_/ITO, 250 nm	3.23 × 10^− 12^	5.11 × 10^− 11^	180	^69^
TiPcCl_2_/FTO, 250 nm	2.12 × 10^− 12^	3.51 × 10^− 11^	204	^69^
TiPcCl_2_/Quartz, 800 nm	1.51 × 10^− 14^	3.03 × 10^− 14^	202.8	^65^
B-subPcCl/glass, 100 nm	~ 2 × 10^− 13^	~ 4 × 10^− 12^	-	^70^
B-subPcCl/polyacetate, 100 nm	~ 2.5 × 10^− 12^	~ 6 × 10^− 11^	-	^71^
(4TFEO) 4-ZnPc/Quartz, 100 nm	-	-	120	^3^
GaPcCl/ Quartz, 200 nm	5.9 × 10^− 12^	1.1 × 10^− 11^	-	^72^
GaPcCl/ Quartz, 151 nm	33.6 × 10^− 12^	41.1 × 10^− 11^	-	^73^
GaPcCl/ Quartz, 98 nm	24.46 × 10^− 12^	31.21 × 10^− 11^	-	^73^
AlPcCl/Quartz, 115 nm	8.21 × 10^− 13^	-	-	^74^
β-H_2_Pc/Quartz, 158 nm	72.637 × 10^− 13^	10.569 × 10^− 11^	-	^75^
β-H_2_Pc/Quartz, 152 nm	40.5 × 10^− 13^	6.68 × 10^− 11^	-	^76^
β-H_2_Pc/Quartz, 1598 nm	18.89 × 10^− 13^	39.24 × 10^− 11^	-	^77^

The nonlinear optical parameters ($\:{\chi\:}^{\left(3\right)}$, $\:{n}_{2}$ and βc) are calculated from semi-empirical equations (Eqs. 9–11), which are commonly employed for organic thin films because they are easy to apply and give good results. These methods, on the other hand, presume that the electronic response is the same everywhere and don’t take into consideration local field effects or individual electronic transitions. Plasmonic enhancements or aggregation-induced nonlinearities, as seen in TiN/PVA nanocomposites^82^ are not included in this discussion. Even with these constraints, the computed values match up with data from similar phthalocyanine systems (see Table 6).

## Conclusions

In conclusion, theoretical and experimental investigations of the structural and optical properties of ZnTTBPc were studied. DFT was used to find the optimized geometrical structure, Mulliken charge analysis, molecular electrostatic potential (MEP) map of the ZnTTBPc molecule. Moreover, HOMO-LUMO and NLO properties such as polarizability and hyperpolarizability were determined. The spin coating process was used to successfully deposit ZnTTBPc thin layer. The XRD patterns demonstrated the amorphous nature of ZnTTBPc powder and thin films. The ZnTTBPc thin film’s FESEM images showed the material’s nanostructure characteristics. The agreement between the powder and thin film Raman spectra confirmed that spin-coating method had ability to produce high quality ZnTTBPc thin films. The energy band gaps of 1.42 and 2.25 eV were employed to designate the Q and B bands, respectively. Reflective index dispersion characteristics in a normal dispersion region were examined using a single oscillator model. Analyzing the ZnTTBPc thin film’s nonlinear optical properties show that it is ideal for many applications that need a strong response. The findings of this study indicated that the ZnTTBPc thin film had considerable potential as a candidate for photonic and optoelectronic devices, due to its high non-linear optical characteristics.

## Supplementary Information

Below is the link to the electronic supplementary material.

Supplementary Material 1

## Data Availability

All data generated or analyzed during this study are included in the manuscript and or supplementary. Additional data or data files can be provided by the corresponding author upon request.
