# Supplementary material for: Structural and optical properties of spin coated ZnTTBPc thin films for optoelectronics via DFT and experiments
Source: Sci Rep. 2025 Oct 17;15:36301. doi: 10.1038/s41598-025-21726-8 (PMC12534632; doi:10.1038/s41598-025-21726-8)
Supplement: Supplementary file 1 — Supplementary Material 1 [file 41598_2025_21726_MOESM1_ESM.pdf]

Structural, Optical, and Nonlinear Properties of Spin-Coated ZnTTBPc Thin Films: A Combined DFT and Experimental Study for Optoelectronic Applications

Mostafa Saad Ebied[1], Sahar Elnobi[2], Amr Attia Abuelwafa[1*]

[1]Nano and Thin film lab. Physics Department, Faculty of Science, South Valley University, Qena 83523, Egypt.

[2]Physics Department, Faculty of Science, South Valley University, Qena 83523, Egypt

***Corresponding author e-mail: amr.abuelwafa@sci.svu.edu.eg (Amr Attia Abuelwafa)**

Table S 1: Bond Lengths of optimized geometry of ZnTTBc molecule.

Bond length (Å)		Bond lengt (Å)		Bond length(Å)		Bond length (Å)	
Zn1-N2	1.99	C19-C23	1.4064	C36-C40	1.4184	C49-H80	1.0939
Zn1-N3	1.99	C19-C27	1.3957	C36-C44	1.54	C49-H81	1.0957
Zn1-N4	1.99	C20-C25	1.4064	C37-C41	1.4184	C50-H82	1.0947
Zn1-N5	1.99	C20-C28	1.3957	C37-C45	1.54	C50-H83	1.0946
N2-C10	1.3733	C21-C24	1.4064	C38-H66	1.0856	C50-H84	1.0947
N2-C14	1.3738	C21-C29	1.3957	C39-H67	1.0856	C51-H85	1.0957
N3-C11	1.3733	C22-C30	1.3951	C40-H68	1.0856	C51-H86	1.0939
N3-C15	1.3738	C23-C31	1.3951	C41-H69	1.0856	C51-H87	1.0948
N4-C12	1.3733	C24-C33	1.3951	C42-C46	1.5475	C52-H88	1.0957
N4-C17	1.3738	C25-C32	1.3951	C42-C47	1.5475	C52-H89	1.0939
N5-C13	1.3733	C26-C34	1.3993	C42-C48	1.5402	C52-H90	1.0948
N5-C16	1.3738	C26-H58	1.0828	C43-C49	1.5475	C53-H91	1.0947
N6-C10	1.3302	C27-C35	1.3993	C43-C50	1.5402	C53-H92	1.0946
N6-C16	1.3304	C27-H59	1.0828	C43-C51	1.5475	C53-H93	1.0947
N7-C12	1.3302	C28-C36	1.3993	C44-C52	1.5475	C54-H94	1.0957
N7-C14	1.3304	C28-H60	1.0828	C44-C53	1.5402	C54-H95	1.0939
N8-C11	1.3302	C29-C37	1.3993	C44-C54	1.5475	C54-H96	1.0948
N8-C17	1.3304	C29-H61	1.0828	C45-C55	1.5402	C55-H97	1.0947
N9-C13	1.3302	C30-C38	1.3907	C45-C56	1.5475	C55-H98	1.0947
N9-C15	1.3304	C30-H62	1.0851	C45-C57	1.5475	C55-H99	1.0946
C10-C18	1.4612	C31-C39	1.3907	C46-H70	1.0939	C56-H100	1.0957
C11-C19	1.4612	C31-H63	1.0851	C46-H71	1.0948	C56-H101	1.0939
C12-C20	1.4612	C32-C40	1.3907	C46-H72	1.0957	C56-H102	1.0948
C13-C21	1.4612	C32-H64	1.0851	C47-H73	1.0939	C57-H103	1.0939
C14-C22	1.4591	C33-C41	1.3907	C47-H74	1.0948	C57-H104	1.0957
C15-C23	1.4591	C33-H65	1.0851	C47-H75	1.0957	C57-H105	1.0948
C16-C24	1.4591	C34-C38	1.4184	C48-H76	1.0947		
C17-C25	1.4591	C34-C42	1.54	C48-H77	1.0947		
C18-C22	1.4064	C35-C39	1.4184	C48-H78	1.0946		
C18-C26	1.3957	C35-C43	1.54	C49-H79	1.0948		

Table S 2: Bond Angles of optimized geometry of ZnTTBc molecule.

Bond Angle (□)		Bond Angle (□)		Bond Angle (□)	
N2-Zn1-N4	90	C20-C28-H60	119.0016	H73-C47-H75	107.6604
N2-Zn1-N5	90	C36-C28-H60	121.8287	H74-C47-H75	108.0748
N3-Zn1-N4	90	C21-C29-C37	119.1697	C42-C48-H76	111.8565
N3-Zn1-N5	90	C21-C29-H61	119.0016	C42-C48-H77	111.8565
Zn1-N2-C10	125.3251	C37-C29-H61	121.8287	C42-C48-H78	109.6276
Zn1-N2-C14	125.3251	C22-C30-C38	117.9643	H76-C48-H77	108.218
C10-N2-C14	109.3497	C22-C30-H62	120.759	H76-C48-H78	107.5478
Zn1-N3-C11	125.3251	C38-C30-H62	121.2766	H77-C48-H78	107.5478
Zn1-N3-C15	125.3251	C23-C31-C39	117.9643	C43-C49-H79	110.9406
C11-N3-C15	109.3497	C23-C31-H63	120.759	C43-C49-H80	111.9765
Zn1-N4-C12	125.3251	C39-C31-H63	121.2766	C43-C49-H81	110.2748
Zn1-N4-C17	125.3252	C25-C32-C40	117.9643	H79-C49-H80	107.7609
C12-N4-C17	109.3497	C25-C32-H64	120.759	H79-C49-H81	108.0748
Zn1-N5-C13	125.3251	C40-C32-H64	121.2766	H80-C49-H81	107.6604
Zn1-N5-C16	125.3251	C24-C33-C41	117.9643	C43-C50-H82	111.8565
C13-N5-C16	109.3497	C24-C33-H65	120.759	C43-C50-H83	109.6276
C10-N6-C16	124.3191	C41-C33-H65	121.2766	C43-C50-H84	111.8565
C12-N7-C14	124.3191	C26-C34-C38	118.3606	H82-C50-H83	107.5478
C11-N8-C17	124.3191	C26-C34-C42	122.4471	H82-C50-H84	108.218
C13-N9-C15	124.3191	C38-C34-C42	119.1922	H83-C50-H84	107.5478
N2-C10-N6	127.5285	C27-C35-C39	118.3606	C43-C51-H85	110.2748
N2-C10-C18	108.7422	C27-C35-C43	122.4472	C43-C51-H86	111.9765
N6-C10-C18	123.7293	C39-C35-C43	119.1922	C43-C51-H87	110.9406
N3-C11-N8	127.5285	C28-C36-C40	118.3606	H85-C51-H86	107.6604
N3-C11-C19	108.7422	C28-C36-C44	122.4472	H85-C51-H87	108.0748
N8-C11-C19	123.7293	C40-C36-C44	119.1922	H86-C51-H87	107.7609
N4-C12-N7	127.5285	C29-C37-C41	118.3606	C44-C52-H88	110.2748
N4-C12-C20	108.7422	C29-C37-C45	122.4471	C44-C52-H89	111.9765
N7-C12-C20	123.7293	C41-C37-C45	119.1923	C44-C52-H90	110.9406
N5-C13-N9	127.5285	C30-C38-C34	122.8098	H88-C52-H89	107.6604
N5-C13-C21	108.7422	C30-C38-H66	118.2512	H88-C52-H90	108.0748
N9-C13-C21	123.7293	C34-C38-H66	118.939	H89-C52-H90	107.7609
N2-C14-N7	127.5021	C31-C39-C35	122.8099	C44-C53-H91	111.8565
N2-C14-C22	108.6812	C31-C39-H67	118.2512	C44-C53-H92	109.6276
N7-C14-C22	123.8166	C35-C39-H67	118.9389	C44-C53-H93	111.8565
N3-C15-N9	127.5021	C32-C40-C36	122.8098	H91-C53-H92	107.5478
N3-C15-C23	108.6813	C32-C40-H68	118.2512	H91-C53-H93	108.218
N9-C15-C23	123.8166	C36-C40-H68	118.9389	H92-C53-H93	107.5478
N5-C16-N6	127.5021	C33-C41-C37	122.8098	C44-C54-H94	110.2748
N5-C16-C24	108.6813	C33-C41-H69	118.2512	C44-C54-H95	111.9765
N6-C16-C24	123.8166	C37-C41-H69	118.939	C44-C54-H96	110.9406
N4-C17-N8	127.5021	C34-C42-C46	109.4619	H94-C54-H95	107.6604
N4-C17-C25	108.6812	C34-C42-C47	109.4619	H94-C54-H96	108.0748
N8-C17-C25	123.8166	C34-C42-C48	112.2638	H95-C54-H96	107.7609
C10-C18-C22	106.4967	C46-C42-C47	109.3616	C45-C55-H97	111.8565
C10-C18-C26	131.9519	C46-C42-C48	108.1204	C45-C55-H98	111.8564
C22-C18-C26	121.5514	C47-C42-C48	108.1203	C45-C55-H99	109.6276
C11-C19-C23	106.4967	C35-C43-C49	109.4619	H97-C55-H98	108.218
C11-C19-C27	131.952	C35-C43-C50	112.2639	H97-C55-H99	107.5478
C23-C19-C27	121.5514	C35-C43-C51	109.4618	H98-C55-H99	107.5479
C12-C20-C25	106.4967	C49-C43-C50	108.1203	C45-C56-H100	110.2748
C12-C20-C28	131.9519	C49-C43-C51	109.3615	C45-C56-H101	111.9766
C25-C20-C28	121.5514	C50-C43-C51	108.1203	C45-C56-H102	110.9406

C13-C21-C24	106.4967	C36-C44-C52	109.4619	H100-C56-H101	107.6604
C13-C21-C29	131.9519	C36-C44-C53	112.2639	H100-C56-H102	108.0748
C24-C21-C29	121.5514	C36-C44-C54	109.4618	H101-C56-H102	107.7609
C14-C22-C18	106.7301	C52-C44-C53	108.1203	C45-C57-H103	111.9766
C14-C22-C30	133.1257	C52-C44-C54	109.3615	C45-C57-H104	110.2748
C18-C22-C30	120.1442	C53-C44-C54	108.1203	C45-C57-H105	110.9407
C15-C23-C19	106.7301	C37-C45-C55	112.2636	H103-C57-H104	107.6604
C15-C23-C31	133.1257	C37-C45-C56	109.4619	H103-C57-H105	107.7609
C19-C23-C31	120.1442	C37-C45-C57	109.462	H104-C57-H105	108.0748
C16-C24-C21	106.7301	C55-C45-C56	108.1204		
C16-C24-C33	133.1257	C55-C45-C57	108.1203		
C21-C24-C33	120.1442	C56-C45-C57	109.3616		
C17-C25-C20	106.7301	C42-C46-H70	111.9766		
C17-C25-C32	133.1257	C42-C46-H71	110.9406		
C20-C25-C32	120.1442	C42-C46-H72	110.2748		
C18-C26-C34	119.1697	H70-C46-H71	107.7609		
C18-C26-H58	119.0016	H70-C46-H72	107.6604		
C34-C26-H58	121.8287	H71-C46-H72	108.0748		
C19-C27-C35	119.1697	C42-C47-H73	111.9766		
C19-C27-H59	119.0016	C42-C47-H74	110.9406		
C35-C27-H59	121.8287	C42-C47-H75	110.2748		
C20-C28-C36	119.1697	H73-C47-H74	107.7609		

Table S 3: Dihedral angles of optimized geometry of ZnTTBc molecule.

Dihedral Angle (□)		Dihedral Angle (□)		Dihedral Angle (□)	
N4-Zn1-N2-C10	179.9997	C27-C19-C23-C31	0	C48-C42-C46-H70	177.4833
N4-Zn1-N2-C14	-0.001	C11-C19-C27-C35	180	C48-C42-C46-H71	-62.0765
N5-Zn1-N2-C10	0.001	C11-C19-C27-H59	0	C48-C42-C46-H72	57.6267
N5-Zn1-N2-C14	-179.9997	C23-C19-C27-C35	0	C34-C42-C47-H73	59.9396
C10-N2-N3-C11	179.9998	C23-C19-C27-H59	179.9999	C34-C42-C47-H74	-60.5007
C10-N2-N3-C15	0	C12-C20-C25-C17	0	C34-C42-C47-H75	179.7961
C14-N2-N3-C11	0	C12-C20-C25-C32	180	C46-C42-C47-H73	-59.9763
C14-N2-N3-C15	-179.9998	C28-C20-C25-C17	-180	C46-C42-C47-H74	179.5834
N4-Zn1-N3-C11	0.001	C28-C20-C25-C32	0	C46-C42-C47-H75	59.8802
N4-Zn1-N3-C15	-179.9997	C12-C20-C28-C36	180	C48-C42-C47-H73	-177.4831
N5-Zn1-N3-C11	179.9997	C12-C20-C28-H60	0	C48-C42-C47-H74	62.0766
N5-Zn1-N3-C15	-0.001	C25-C20-C28-C36	0	C48-C42-C47-H75	-57.6266
N2-Zn1-N4-C12	0.001	C25-C20-C28-H60	180	C34-C42-C48-H76	-60.795
N2-Zn1-N4-C17	-179.9997	C13-C21-C24-C16	0	C34-C42-C48-H77	60.7944
N3-Zn1-N4-C12	179.9997	C13-C21-C24-C33	180	C34-C42-C48-H78	-180.0003
N3-Zn1-N4-C17	-0.001	C29-C21-C24-C16	-180	C46-C42-C48-H76	60.054
C12-N4-N5-C13	179.9998	C29-C21-C24-C33	0	C46-C42-C48-H77	-178.3566
C12-N4-N5-C16	0	C13-C21-C29-C37	180	C46-C42-C48-H78	-59.1513
C17-N4-N5-C13	0	C13-C21-C29-H61	0	C47-C42-C48-H76	178.3561
C17-N4-N5-C16	-179.9998	C24-C21-C29-C37	0	C47-C42-C48-H77	-60.0546
N2-Zn1-N5-C13	179.9997	C24-C21-C29-H61	179.9999	C47-C42-C48-H78	59.1507
N2-Zn1-N5-C16	-0.001	C14-C22-C30-C38	180	C35-C43-C49-H79	60.5008
N3-Zn1-N5-C13	0.001	C14-C22-C30-H62	0	C35-C43-C49-H80	-59.9395
N3-Zn1-N5-C16	-179.9997	C18-C22-C30-C38	0	C35-C43-C49-H81	-179.796
Zn1-N2-C10-N6	-7.00E-04	C18-C22-C30-H62	180	C50-C43-C49-H79	-62.0767
Zn1-N2-C10-C18	179.9994	C15-C23-C31-C39	-180	C50-C43-C49-H80	177.4831
C14-N2-C10-N6	179.9999	C15-C23-C31-H63	0	C50-C43-C49-H81	57.6265
C14-N2-C10-C18	0	C19-C23-C31-C39	0	C51-C43-C49-H79	-179.5835
Zn1-N2-C14-N7	7.00E-04	C19-C23-C31-H63	-180	C51-C43-C49-H80	59.9763
Zn1-N2-C14-C22	-179.9994	C16-C24-C33-C41	-180	C51-C43-C49-H81	-59.8803
C10-N2-C14-N7	-179.9999	C16-C24-C33-H65	0	C35-C43-C50-H82	60.7954
C10-N2-C14-C22	0	C21-C24-C33-C41	0	C35-C43-C50-H83	180.0007
Zn1-N3-C11-N8	-7.00E-04	C21-C24-C33-H65	-180	C35-C43-C50-H84	-60.794
Zn1-N3-C11-C19	179.9994	C17-C25-C32-C40	-180	C49-C43-C50-H82	-178.3556
C15-N3-C11-N8	179.9999	C17-C25-C32-H64	0	C49-C43-C50-H83	-59.1503
C15-N3-C11-C19	0	C20-C25-C32-C40	0	C49-C43-C50-H84	60.055
Zn1-N3-C15-N9	7.00E-04	C20-C25-C32-H64	-180	C51-C43-C50-H82	-60.0536
Zn1-N3-C15-C23	-179.9994	C18-C26-C34-C38	0	C51-C43-C50-H83	59.1517
C11-N3-C15-N9	-179.9999	C18-C26-C34-C42	-180	C51-C43-C50-H84	178.357
C11-N3-C15-C23	0	H58-C26-C34-C38	180	C35-C43-C51-H85	179.7957
Zn1-N4-C12-N7	-7.00E-04	H58-C26-C34-C42	0	C35-C43-C51-H86	59.9392
Zn1-N4-C12-C20	179.9994	C19-C27-C35-C39	0	C35-C43-C51-H87	-60.5011
C17-N4-C12-N7	179.9999	C19-C27-C35-C43	180	C49-C43-C51-H85	59.88
C17-N4-C12-C20	0	H59-C27-C35-C39	-179.9999	C49-C43-C51-H86	-59.9766
Zn1-N4-C17-N8	7.00E-04	H59-C27-C35-C43	1.00E-04	C49-C43-C51-H87	179.5831
Zn1-N4-C17-C25	-179.9994	C20-C28-C36-C40	0	C50-C43-C51-H85	-57.6268
C12-N4-C17-N8	-179.9999	C20-C28-C36-C44	180	C50-C43-C51-H86	-177.4834
C12-N4-C17-C25	0	H60-C28-C36-C40	-180	C50-C43-C51-H87	62.0764
Zn1-N5-C13-N9	-7.00E-04	H60-C28-C36-C44	0	C36-C44-C52-H88	-179.796
Zn1-N5-C13-C21	179.9994	C21-C29-C37-C41	0	C36-C44-C52-H89	-59.9394
C16-N5-C13-N9	179.9999	C21-C29-C37-C45	180.0001	C36-C44-C52-H90	60.5009
C16-N5-C13-C21	0	H61-C29-C37-C41	-179.9999	C53-C44-C52-H88	57.6266

Zn1-N5-C16-N6	8.00E-04
Zn1-N5-C16-C24	-179.9994
C13-N5-C16-N6	-179.9999
C13-N5-C16-C24	0
C16-N6-C10-N2	-1.00E-04
C16-N6-C10-C18	179.9998
C10-N6-C16-N5	0
C10-N6-C16-C24	-179.9998
C14-N7-C12-N4	0
C14-N7-C12-C20	179.9999
C12-N7-C14-N2	0
C12-N7-C14-C22	-179.9998
C17-N8-C11-N3	0
C17-N8-C11-C19	179.9998
C11-N8-C17-N4	0
C11-N8-C17-C25	-179.9998
C15-N9-C13-N5	0
C15-N9-C13-C21	179.9999
C13-N9-C15-N3	0
C13-N9-C15-C23	-179.9998
N2-C10-C18-C22	0
N2-C10-C18-C26	180
N6-C10-C18-C22	-179.9999
N6-C10-C18-C26	1.00E-04
N3-C11-C19-C23	0
N3-C11-C19-C27	180
N8-C11-C19-C23	-179.9999
N8-C11-C19-C27	1.00E-04
N4-C12-C20-C25	0
N4-C12-C20-C28	180
N7-C12-C20-C25	-179.9999
N7-C12-C20-C28	1.00E-04
N5-C13-C21-C24	0
N5-C13-C21-C29	180
N9-C13-C21-C24	-179.9999
N9-C13-C21-C29	0
N2-C14-C22-C18	0
N2-C14-C22-C30	-180
N7-C14-C22-C18	179.9999
N7-C14-C22-C30	-1.00E-04
N3-C15-C23-C19	0
N3-C15-C23-C31	-180
N9-C15-C23-C19	179.9999
N9-C15-C23-C31	-1.00E-04
N5-C16-C24-C21	0
N5-C16-C24-C33	-180
N6-C16-C24-C21	179.9999
N6-C16-C24-C33	-1.00E-04
N4-C17-C25-C20	0
N4-C17-C25-C32	-180
N8-C17-C25-C20	179.9999
N8-C17-C25-C32	-1.00E-04
C10-C18-C22-C14	0
C10-C18-C22-C30	-180
C26-C18-C22-C14	-180
C26-C18-C22-C30	0

H61-C29-C37-C45	1.00E-04
C22-C30-C38-C34	0
C22-C30-C38-H66	180
H62-C30-C38-C34	180
H62-C30-C38-H66	0
C23-C31-C39-C35	0
C23-C31-C39-H67	-180
H63-C31-C39-C35	180
H63-C31-C39-H67	0
C25-C32-C40-C36	0
C25-C32-C40-H68	-180
H64-C32-C40-C36	180
H64-C32-C40-H68	0
C24-C33-C41-C37	0
C24-C33-C41-H69	-180.0001
H65-C33-C41-C37	-180
H65-C33-C41-H69	-1.00E-04
C26-C34-C38-C30	0
C26-C34-C38-H66	-180
C42-C34-C38-C30	180
C42-C34-C38-H66	0
C26-C34-C42-C46	-120.0731
C26-C34-C42-C47	120.0725
C26-C34-C42-C48	-3.00E-04
C38-C34-C42-C46	59.9269
C38-C34-C42-C47	-59.9275
C38-C34-C42-C48	-180.0003
C27-C35-C39-C31	0
C27-C35-C39-H67	180
C43-C35-C39-C31	-180
C43-C35-C39-H67	0
C27-C35-C43-C49	-120.0721
C27-C35-C43-C50	8.00E-04
C27-C35-C43-C51	120.0737
C39-C35-C43-C49	59.9279
C39-C35-C43-C50	180.0008
C39-C35-C43-C51	-59.9263
C28-C36-C40-C32	0
C28-C36-C40-H68	180
C44-C36-C40-C32	-180
C44-C36-C40-H68	0
C28-C36-C44-C52	-120.0724
C28-C36-C44-C53	5.00E-04
C28-C36-C44-C54	120.0734
C40-C36-C44-C52	59.9276
C40-C36-C44-C53	180.0005
C40-C36-C44-C54	-59.9266
C29-C37-C41-C33	0
C29-C37-C41-H69	180.0001
C45-C37-C41-C33	-180.0001
C45-C37-C41-H69	0
C29-C37-C45-C55	0.0015
C29-C37-C45-C56	120.0743
C29-C37-C45-C57	-120.0712
C41-C37-C45-C55	180.0016
C41-C37-C45-C56	-59.9257

C53-C44-C52-H89	177.4831
C53-C44-C52-H90	-62.0766
C54-C44-C52-H88	-59.8802
C54-C44-C52-H89	59.9763
C54-C44-C52-H90	-179.5834
C36-C44-C53-H91	-60.7942
C36-C44-C53-H92	180.0004
C36-C44-C53-H93	60.7951
C52-C44-C53-H91	60.0548
C52-C44-C53-H92	-59.1506
C52-C44-C53-H93	-178.3558
C54-C44-C53-H91	178.3568
C54-C44-C53-H92	59.1514
C54-C44-C53-H93	-60.0539
C36-C44-C54-H94	179.7958
C36-C44-C54-H95	59.9392
C36-C44-C54-H96	-60.501
C52-C44-C54-H94	59.88
C52-C44-C54-H95	-59.9765
C52-C44-C54-H96	179.5832
C53-C44-C54-H94	-57.6268
C53-C44-C54-H95	-177.4833
C53-C44-C54-H96	62.0764
C37-C45-C55-H97	60.796
C37-C45-C55-H98	-60.7933
C37-C45-C55-H99	180.0013
C56-C45-C55-H97	-60.0529
C56-C45-C55-H98	178.3578
C56-C45-C55-H99	59.1524
C57-C45-C55-H97	-178.355
C57-C45-C55-H98	60.0556
C57-C45-C55-H99	-59.1497
C37-C45-C56-H100	179.7959
C37-C45-C56-H101	59.9394
C37-C45-C56-H102	-60.5009
C55-C45-C56-H100	-57.6269
C55-C45-C56-H101	-177.4835
C55-C45-C56-H102	62.0762
C57-C45-C56-H100	59.8799
C57-C45-C56-H101	-59.9766
C57-C45-C56-H102	179.5831
C37-C45-C57-H103	-59.9399
C37-C45-C57-H104	-179.7965
C37-C45-C57-H105	60.5004
C55-C45-C57-H103	177.4829
C55-C45-C57-H104	57.6263
C55-C45-C57-H105	-62.0768
C56-C45-C57-H103	59.976
C56-C45-C57-H104	-59.8805
C56-C45-C57-H105	-179.5837

C10-C18-C26-C34	-180	C41-C37-C45-C57	59.9288
C10-C18-C26-H58	0	C34-C42-C46-H70	-59.9394
C22-C18-C26-C34	0	C34-C42-C46-H71	60.5008
C22-C18-C26-H58	180	C34-C42-C46-H72	-179.796
C11-C19-C23-C15	0	C47-C42-C46-H70	59.9764
C11-C19-C23-C31	180	C47-C42-C46-H71	-179.5833
C27-C19-C23-C15	-180	C47-C42-C46-H72	-59.8801

Table S 4: Optimized structure of ZnTTBPc molecule.

Zn	0	0	0
N	1.9164	0.5361	0
N	-1.9164	-0.5361	0
N	0.5361	-1.9164	0
N	-0.5361	1.9164	0
N	1.6592	2.9475	0
N	2.9475	-1.6592	0
N	-1.6592	-2.9475	0
N	-2.9475	1.6592	0
C	2.3793	1.829	0
C	-2.3793	-1.829	0
C	1.829	-2.3793	0
C	-1.829	2.3793	0
C	2.9833	-0.3294	0
C	-2.9833	0.3294	0
C	0.3294	2.9833	0
C	-0.3294	-2.9833	0
C	3.8403	1.8047	0
C	-3.8403	-1.8047	0
C	1.8047	-3.8403	0
C	-1.8047	3.8403	0
C	4.2171	0.4497	0
C	-4.2171	-0.4497	0
C	-0.4497	4.2171	0
C	0.4497	-4.2171	0
C	4.7905	2.827	0
C	-4.7905	-2.827	0
C	2.827	-4.7905	0
C	-2.827	4.7905	0
C	5.5671	0.098	0
C	-5.5671	-0.098	0
C	0.098	-5.5671	0
C	-0.098	5.5671	0
C	6.1498	2.4947	0
C	-6.1498	-2.4947	0
C	2.4947	-6.1498	0
C	-2.4947	6.1498	0
C	6.5079	1.1222	0
C	-6.5079	-1.1222	0
C	1.1222	-6.5079	0
C	-1.1222	6.5079	0
C	7.261	3.5609	0
C	-7.261	-3.5609	0
C	3.5609	-7.261	0
C	-3.5609	7.261	0
C	8.1392	3.3903	-1.2627
C	8.1392	3.3903	1.2627
C	6.6952	4.9934	0
C	-8.1392	-3.3903	-1.2627
C	-6.6952	-4.9934	0
C	-8.1393	-3.3903	1.2627
C	3.3903	-8.1392	-1.2627
C	4.9934	-6.6952	0
C	3.3903	-8.1393	1.2627

C	-4.9934	6.6952	0
C	-3.3903	8.1393	1.2627
C	-3.3903	8.1392	-1.2627
H	4.4542	3.8563	0
H	-4.4542	-3.8563	0
H	3.8563	-4.4542	0
H	-3.8563	4.4542	0
H	5.8691	-0.9443	0
H	-5.8691	0.9443	0
H	-0.9443	-5.8691	0
H	0.9443	5.8691	0
H	7.5597	0.8538	0
H	-7.5597	-0.8538	0
H	0.8538	-7.5597	0
H	-0.8538	7.5597	0
H	8.6106	2.404	-1.3033
H	7.5438	3.5135	-2.1731
H	8.9374	4.1409	-1.2743
H	8.6106	2.404	1.3033
H	7.5438	3.5135	2.1731
H	8.9374	4.1409	1.2743
H	6.0844	5.1904	-0.8869
H	6.0844	5.1904	0.8869
H	7.5191	5.7142	0
H	-7.5437	-3.5135	-2.1731
H	-8.6106	-2.404	-1.3033
H	-8.9374	-4.1409	-1.2743
H	-6.0844	-5.1904	0.8869
H	-7.5191	-5.7142	0
H	-6.0844	-5.1904	-0.8869
H	-8.9374	-4.1409	1.2743
H	-8.6106	-2.404	1.3032
H	-7.5438	-3.5135	2.173
H	4.1409	-8.9374	-1.2743
H	2.404	-8.6106	-1.3033
H	3.5135	-7.5437	-2.1731
H	5.1904	-6.0844	-0.8869
H	5.7142	-7.5191	0
H	5.1904	-6.0844	0.8869
H	4.1409	-8.9374	1.2743
H	2.404	-8.6106	1.3032
H	3.5135	-7.5438	2.1731
H	-5.1904	6.0844	0.8869
H	-5.1904	6.0843	-0.8868
H	-5.7142	7.5191	0
H	-4.1409	8.9374	1.2743
H	-2.404	8.6106	1.3032
H	-3.5135	7.5438	2.1731
H	-2.4041	8.6106	-1.3033
H	-4.1409	8.9374	-1.2744
H	-3.5136	7.5437	-2.1731

Table S 5: Calculated vertical transition energy E_{ver} ,maximum absorption wavelengths (λ_{max}), oscillator strengths (f) and the orbitals involved in the transitions for ZnTTBPc Molecule.

No	E_{ver}(eV)	λ_{max} (nm)	f	Symmetry	Major contribs
1	2.054	603.62314	0.5621	Singlet-A	HOMO->LUMO (93%)
2	2.054	603.62314	0.5621	Singlet-A	HOMO->L+1 (93%)
3	3.5023	354.00792	0.0	Singlet-A	H-5->LUMO (93%)
4	3.5023	354.00792	0.0	Singlet-A	H-5->L+1 (93%)
5	3.8116	325.28123	0.0	Singlet-A	H-4->LUMO (44%), H-3->L+1 (44%)
6	3.8248	324.15863	0.0	Singlet-A	H-4->LUMO (42%), H-3->L+1 (42%)
7	3.8944	318.36533	0.0208	Singlet-A	H-9->L+1 (14%), H-2->L+1 (71%)
8	3.8944	318.36533	0.0208	Singlet-A	H-9->LUMO (14%), H-2->LUMO (71%)
9	3.9453	314.25796	0.0	Singlet-A	HOMO->L+2 (78%)
10	4.1205	300.89599	0.0	Singlet-A	H-10->LUMO (88%)
11	4.1205	300.89599	0.0	Singlet-A	H-10->L+1 (88%)
12	4.2097	294.52026	1.3882	Singlet-A	H-9->LUMO (13%), H-1->LUMO (69%)
13	4.2097	294.52026	1.3882	Singlet-A	H-9->L+1 (13%), H-1->L+1 (69%)
14	4.2611	290.96757	0.0	Singlet-A	H-4->L+1 (31%), H-3->LUMO (31%), HOMO->L+2 (17%)
15	4.2804	289.65562	0.0	Singlet-A	HOMO->L+3 (92%)
